# Dentatacid A: An Unprecedented 2, 3-*Seco*-arbor-2, 3-dioic Triterpenoid from the Invasive Plant *Euphorbia dentata*, with Cytotoxicity Effect on Colon Cancer

**DOI:** 10.3390/plants13172533

**Published:** 2024-09-09

**Authors:** Chen-Sen Xu, Yuan-Ling Shao, Qing Li, Yu Zhang, Hong-Wei Wu, Hao-Lin Yu, Yun-Yun Su, Jing Zhang, Chao Wang, Zhi-Xin Liao

**Affiliations:** 1 Department of Pharmaceutical Engineering, School of Chemistry and Chemical Engineering and Jiangsu Province Hi-Tech Key Laboratory for Biomedical Research, Southeast University, Nanjing 211189, China; 2 Changshu Institute for Products Quality Supervision and Inspection, Changshu Measurement and Testing Center, Suzhou 215500, China

**Keywords:** invasive plant, *Euphorbia dentata* Michx., triterpenoid, anti-tumor, colon cancer

## Abstract

*Euphorbia dentata* Michx. is an invasive plant species in China, known for its toxicity and potential to reduce crop yields, posing numerous threats. To gain a deeper understanding of this invasive plant, phytochemical methods were employed to isolate 13 terpenoids (**1**–**11**, **19**, **20**) and 7 sterols (**12**–**18**) from the ethanol extract of *E. dentata*, identifying one new compound and 19 known compounds. Within spectroscopic methods such as NMR, HR-ESI-MS, and ECD, the structures and absolute configurations of these compounds were established. Among them, dentatacid A (**11**) possesses an unprecedented 2, 3-*seco*-arbor-2, 3-dioic skeleton within the potential biosynthetic pathway proposed. Dentatacid A also exhibited excellent anti-proliferative activity against the HT-29 (human colorectal adenocarcinoma) cell line, with an IC_50_ value of 2.64 ± 0.78 μM, which was further confirmed through network pharmacology and molecular docking. This study significantly expands the chemical diversity of *E. dentata* and offers new insights into the resource utilization and management of this invasive plant from the perspective of natural product discovery.

## 1. Introduction

Plant invasion is a global phenomenon associated with human activities and socio-economic driving factors [1]. With the advancement of globalization, plant invasions have increasingly presented negative impacts on human societies [2]. These invasions not only affect the ecological systems of the invaded areas but also pose significant threats to the economic and cultural lives of the local populations. *Euphorbia dentata* Michx., commonly known as the toothed spurge, is an invasive plant species belonging to the genus *Euphorbia* in the family Euphorbiaceae. Originally native to North America, its distribution has expanded to South America [3], Oceania [4], East Asia [4,5], and throughout Europe due to the phenomenon of plant invasion [6]. Research indicates that the white latex produced by *E. dentata* possesses a certain level of toxicity [7]. Moreover, when this species acts as a weed in agricultural fields, such as those growing soybeans, it significantly reduces soybean yields, thereby causing considerable distress to farmers [8,9].

Compared to the recognition of *E. dentata* as an invasive species, many plants from the Euphorbiaceae family and the *Euphorbia* genus have received considerable acclaim in China. Plants of the *Euphorbia* genus, such as Langdu (*E. fischeriana* auct. non Steud.), Jingangzuan (*E. neriifolia* L.), Zeqi (*E. helioscopia* L.), Xusuizi (*E. lathyris* L.), Feiyangcao (*E. hirta* L.), and Dijincao (*E. humifusa* Willd. ex Schltdl.) are widely used in traditional Chinese medicine due to their ability to clear heat and detoxify. Additionally, contemporary methods of separation and extraction have revealed that diterpenoids (such as ingenane type and tigliane type) from the *Euphorbia* genus, representative of the genus, exhibit a rich array of natural product components, including terpenes, sterols, and flavonoids [10,11]. Further pharmacological research has demonstrated that natural products derived from plants in the *Euphorbia* genus exhibit exceptional anti-inflammatory, antioxidant, antiviral, and cytotoxic activities [10,12,13]. Therefore, investigating the chemical constituents of *E. dentata* through phytochemical approaches could not only aid in the discovery of novel bioactive molecules but also contribute to the research on the resource utilization of invasive plants, thereby mitigating issues related to biological invasion.

**Figure 1:**
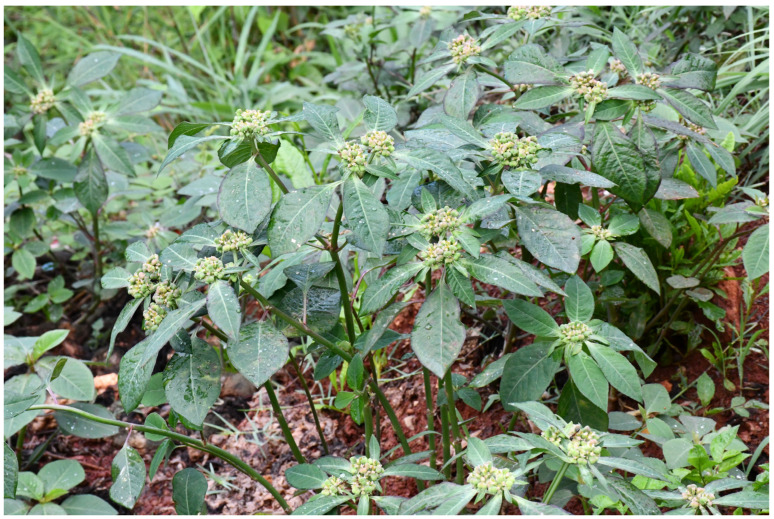
*Euphorbia dentata* Michx. in Yunnan Province, People’s Republic of China.

**Figure 2:**
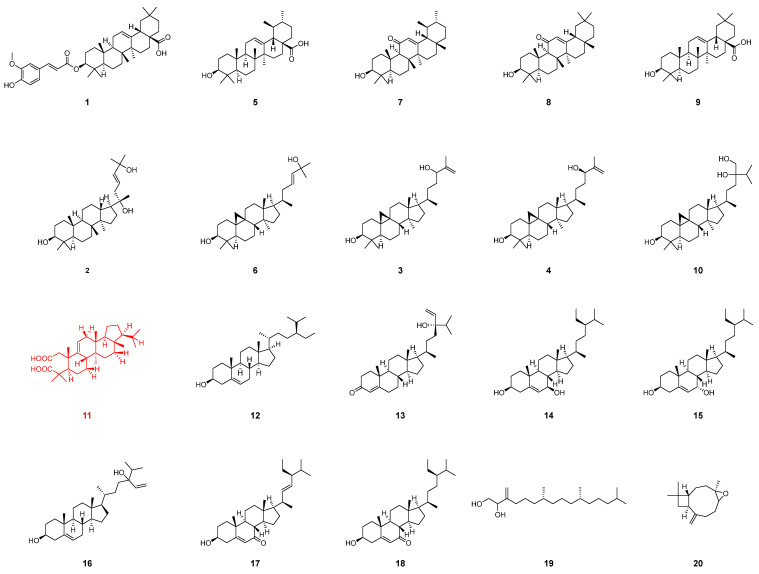
Structures of compounds **1**–**20**.

In previous studies, An et al. specifically selected the toxic white latex of *E. dentata* as the subject of their research, identifying several sesquiterpenes with significant activity against *Helicoverpa armigera* [7]. This study, however, approaches the investigation from a different perspective by examining the entire plant of *E. dentata* (as shown in Figure 1). Ultimately, 11 structurally diverse triterpenoids, 7 sterols, a diterpenoid, and a sesquiterpenoid were successfully isolated and identified from *E. dentata* (as shown in Figure 2). Among these, dentatacid A is a novel entity featuring an unprecedented overoxidized 2, 3-seco-arborinane triterpenoid skeleton. The structure of the dentatacid A was elucidated through HR-ESI-MS, 1D/2D NMR spectroscopy, and ECD calculations. Additionally, the cytotoxicity of these compounds against four types of cancer cells, 143B (human osteosarcoma cell line 143B), A549 (human alveolar basal epithelial), HepG2 (human liver carcinoma cell line), and HT-29 (human colorectal adenocarcinoma) cell lines, was assessed using the CCK-8 assay. Furthermore, network pharmacology and molecular docking approaches were employed to further analyze the exceptional cytotoxicity of these compounds.

## 2. Result and Discussion

Dentatacid A (**11**) was obtained as a white powder (chloroform/methanol). Its molecular formula was determined to be C_30_H_48_O_4_ with 7 degrees of unsaturation (DoUs), based on the (+)-HR-ESI-MS ion peak at m/z 495.3441 [M + Na]^+^ (calcd for C_30_H_48_NaO_4_^+^, 495.3446) and the ^13^C-NMR data (as shown in Table 1). Further analysis of the DEPT-135 data (as shown in Figure S5) revealed that **11** contain 8 quaternary carbons, together with 6 CH signals, 8 CH_2_ signals, and 8 CH_3_ signals. The presence of 8 characteristic methyl groups, *δ*_H_ 1.10 (s, 3H, H-24), 1.09 (s, 6H, H-23, 25), 0.85 (d, *J* = 6.5 Hz, 3H, H-30), 0.79 (d, *J* = 6.5 Hz, 3H, H-29), 0.74 (s, 3H, H-26), 0.71 (s, 3H, H-27), and 0.70 (s, 3H, H-28), suggests that the compound is likely a triterpenoid. The signals at *δ*_C_ 145.9 and 114.5 in ^13^C-NMR, along with *δ*_H_ 5.23 (d, J = 5.7 Hz, 1H, H-11), indicate the presence of a double bond within the compound. *δ*_C_ 172.4 and 180.6 reveal the presence of two carbonyl groups. Based on the chemical formula, the compound contains four oxygen atoms. However, no hydroxyl hydrogen signals were observed in the proton spectrum, nor were any oxygen-bonded carbon signals (hydroxyl or ether groups) detected in the carbon spectrum. Considering *δ*_H_ 11.91 (s, 2H, H-2 COOH) and the infrared (IR) absorption bands at 3443.28 cm^−1^, it is inferred that the compound contains two carboxyl groups. From the analysis of unsaturation, the pentacyclic triterpenoid has an unsaturation degree of 5, one double bond accounts for an unsaturation degree of 1, and two carboxyl groups account for an unsaturation degree of 2, giving a total unsaturation degree of 8, which does not match the observed value. It is known that methyl groups in triterpenoids can easily be oxidized to carboxyl groups, whereas ^1^H-NMR data of this compound reveal the presence of 8 methyl groups, none of which are oxidized. Therefore, it is inferred that this compound possesses a rare open-chain triterpenoid skeleton, with ring cleavage sites being unusually over-oxidized to form two carboxyl groups.

In the HMBC spectrum (as shown in Figure S7), the carboxyl carbon signal at *δ*_C_ 172.4 correlates with H-1 and H-5, and the signal at *δ*_C_ 180.6 correlates with H-5, H-23, and H-24, suggesting that the A ring has been oxidatively cleaved, which forms a 2,3-seco triterpenoid. The signal at *δ*_C_ 145.9 correlates with H-1, H-5, and H-25, while the proton signal at *δ*_H_ 5.23 (d, J = 5.7 Hz, 1H, H-11) correlates with C-8, C-10, and C-13, thus confirming C-9 and C-11 are connected by a carbon-carbon double bond. The proton signal at *δ*_H_ 1.09 (s, H-25) correlates with *δ*_C_ 42.0 and *δ*C 145.9, confirming that Me-25 is attached to C-10. The proton signal at *δ*_H_ 0.74 (s, H-26) correlates with C-8 and C-15, confirming that Me-26 is attached to C-14. The proton signal at *δ*_H_ 0.71 (s, H-27) correlates with C-18, and the proton signal at *δ*_H_ 1.59 (d, J = 2.9 Hz, H-12) correlates with C-27, confirming that Me-27 is attached to C-13. The signal at *δ*_C_ 13.8 correlates with H-16, H-18, and H-21, confirming that Me-28 is attached to C-17. The proton signal at *δ*_H_ 1.52 (m, H-18) correlates with C-13, C-17, and C-19, while the signals at *δ*_H_ 1.28 (m, H-19), *δ*_H_ 1.76 (m, H-20), and *δ*_H_ 0.94 (q, J = 9.4 Hz, H-21) all correlate with C-17, confirming that the E ring is a five-membered ring. The signal at *δ*_C_ 59.0 correlates with H-29 and H-30, while the signal at *δ*_C_ 30.3 correlates with H-21, H-29, and H-30.

**Figure 3:**
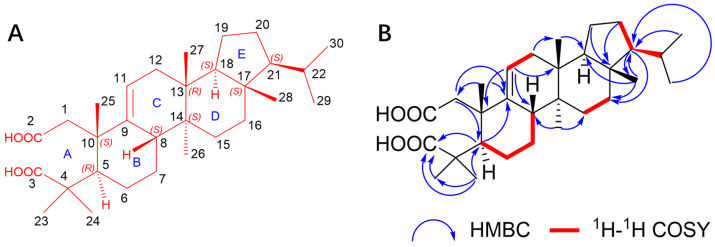
The structure (**A**), the key HMBC, and ^1^H–^1^H COSY correlations (**B**) of **11**.

Additionally, the ^1^H-^1^H COSY spectrum (as shown in Figure 3 and Figure S8) shows correlations between H-21, H-20, and H-22, confirming the presence of an isopropyl group attached to C-21 in the triterpenoid. Concurrently, correlations are observed between H-5/H-6, H-6/H-7, and H-7/H-8, as well as between H-11 and H-12, H-15 and H-16, and H-20 and H-21. These correlations, combined with the aforementioned HMBC data, confirm the linkage of the B, C, D, and E rings.

**Figure 4:**
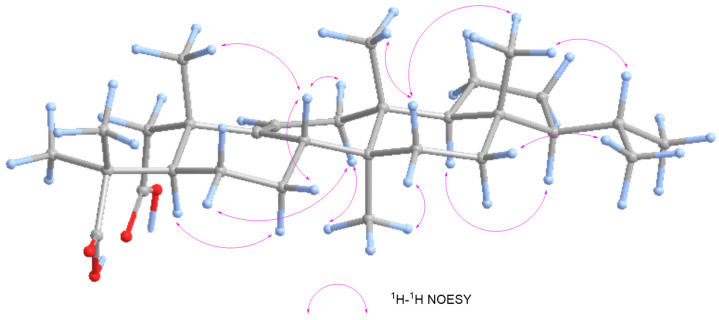
Key NOESY correlations of **11**.

In the ^1^H-^1^H NOESY spectrum (as shown in Figure 4 and Figure S9), H-5 shows correlations with H-7α, H-26 correlates with H-6α/H-12α/H-15α, and H-18 correlates with H-21. Additionally, H-8 correlates with H-7β/H-12β/H-25, H-27 correlates with H-15β, H-28 correlates with H-15β/H-22, and H-30 correlates with H-16β. Based on these correlations, H-8, Me-24, Me-27, and Me-28 are in the same plane and are β-substituents, while H-5, Me-26, H-18, and H-21 are in the same plane and are α-substituents. Thus, the relative configuration of **11** can be determined as an arborinane-type triterpenoid.

**Figure 5:**
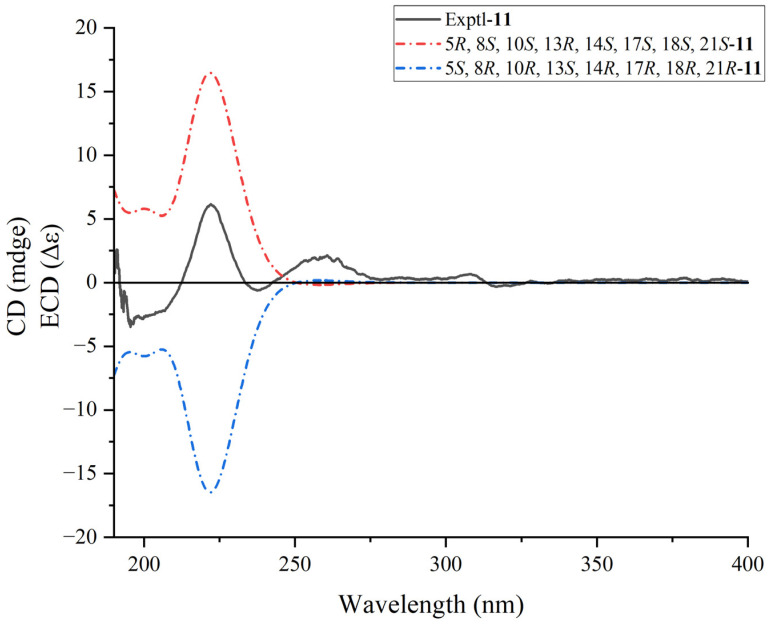
Calculated and experimental ECD spectra for **11**.

Moreover, TDDFT ECD calculation was performed to confirm the absolute configuration of **11** at the B3LYP/6-311++G(2d,2p) level. The ECD curves of **11** showed very similar tendencies in the range 190 to 400 nm (as shown in Figure 5), indicating identical absolute configurations of 5*R*, 8*S*, 10*S*, 13*R*, 14*S*, 17*S*, 18*S*, and 21*S*. Ultimately, **11** has been determined as (5*R*, 8*S*, 10*S*, 13*R*, 14*S*, 17*S*, 18*S*, 21*S*)-2, 3-*seco*-arbor-9-en-2, 3-dioic acid, designated as dentata A.

Unprecedentedly, dentata A is the first-reported arborinane-type triterpenoid with the structure of 2, 3-seco-2, 3-dioic acid, which skeleton is represented by fewer than 30 entries in CAS SciFinder (https://scifinder-n.cas.org/, accessed on 1 August 2024) [14].

In addition to Dentata A, 19 known analogs, including β-Amyrin ferulate (**1**) [15], isofouquierol (**2**) [16], 9,19-Cyclolanost-25-ene-3β,24-diol (**3**) [17], 9,19-Cyclolanost-25-ene-3β,24R-diol (**4**) [17], Ursolic acid (**5**) [18], cycloart-23-ene-3β,25-diol (**6**) [19], 11-Oxo-α-amyrin (**7**) [20], 11-Oxo-β-amyrin (**8**) [21], Oleanolic acid (**9**) [22], 24-methylcycloartane-3β,24,241-triol (**10**) [23], β-sitosterol (**12**) [24], 24-hydroxystigmasta-4,28-dien-3-one (**13**) [25], 7β-hydroxysitosterol (**14**) [26], 7α-hydroxysitosterol (**15**) [26], saringosterol (**16**) [27], 3β-hydroxy-stigmast-5,22-dien-7-one (**17**) [28], 3β-hydroxystigmast-5-en-7-one (**18**) [29], phytyldiol (**19**) [30] and caryophyllene oxide (**20**) [30], were isolated and identified by comparing NMR and MS data with the previously reported (as shown in the NMR data for known compounds from Supplementary Materials). Additionally, 19 known compounds have been isolated from *E. dentata* for the first time.

### 2.1. Hypothetical Biosynthesis for Dentatacid A

**Figure 6:**
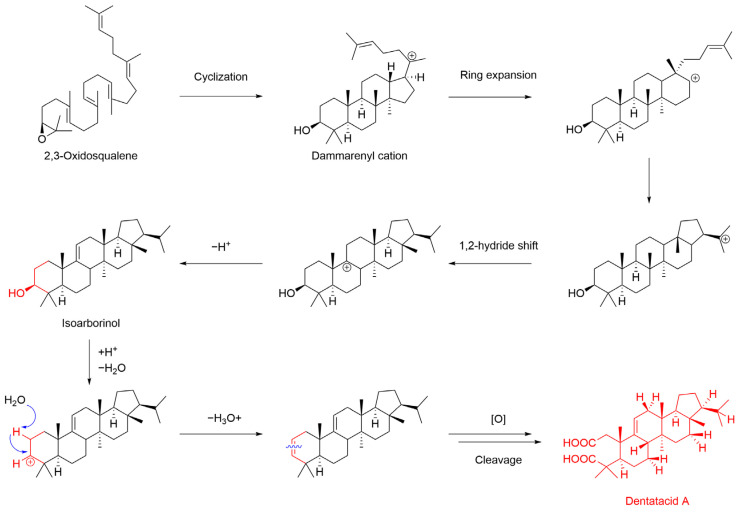
Plausible biogenetic pathways for dentatacid A (**1**).

Due to the novelty of the chemical structure of dentatacid A, we propose a plausible biosynthetic pathway for dentatacid A. 2,3-Oxidosqualene (OSC) is a recognized precursor to triterpenoid derivatives [31]. As shown in Figure 6, OSC undergoes cyclization to form the dammarenyl cation, which, through a series of ring expansions and carbon cation migrations, yields isoarborinol, the arborinane-type triterpenoid skeleton compound [32]. In a potentially aqueous and mildly acidic environment in nature, isoarborinol undergoes dehydration and undergoes an affinity elimination reaction to form a 2,3-en triterpenoid skeleton [33,34]. This is followed by a series of oxidative cleavages and ring openings, ultimately leading to the formation of dentatacid A.

### 2.2. Cytotoxicity Assay

Small molecules derived from natural products have played a crucial role in the discovery of anti-tumor compounds, with triterpenoids bearing carboxyl groups being among the most significant classes of these natural products [35,36]. Our laboratory has been dedicated to the study of the cytotoxicity of natural terpenoids with novel structures [37,38,39,40]. Based on this focus, with HCPT as the control group, the CCK-8 assay is employed to preliminary investigate the cytotoxic effects of 11 triterpenoid compounds on tumor cell lines 143B, A549, HepG2, and HT-29.

In the cytotoxicity assay, all triterpenoids from *E. dentata* exhibited varying degrees of cytotoxicity against the four tumor cell lines, with half of the compounds showing IC_50_ values less than 20 μM (as shown in Table 2). For the A549 cell line, compounds **4**, **5**, **7,** and **8**, along with the novel **11**, demonstrated excellent cytotoxicity, with IC_50_ values ranging from 4.74 to 10.04 μM. Notably, compounds **4** and **11** had IC_50_ values of 4.74 ± 2.61 and 4.85 ± 1.14 μM, respectively, which are very close to the cytotoxicity of HCPT, with an IC_50_ value of 2.89 ± 1.47 μM. However, for the HepG2 and 143B cell lines, the triterpenoids showed less promising cytotoxicity, with many compounds having IC_50_ values greater than 50 μM. The IC_50_ values of **11** were 10.65 ± 2.17 and 18.82 ± 0.60 μM for HepG2 and 143B, respectively. Interestingly, in the cytotoxicity assay against the HT-29 cell line, the triterpenoids exhibited outstanding performance. Compounds **4** and **5** had IC_50_ values of 8.92 ± 2.82 and 5.94 ± 2.13 μM, respectively, while compound **11** showed even more remarkable activity with an IC_50_ value of 2.64 ± 0.78 μM, surpassing HCPT’s IC_50_ value of 2.70 ± 0.16 μM for HT-29.

However, due to experimental constraints, the effects of these compounds on non-tumorigenic cells require further in-depth investigation.

### 2.3. Bioinformatics Analysis

**Figure 7:**
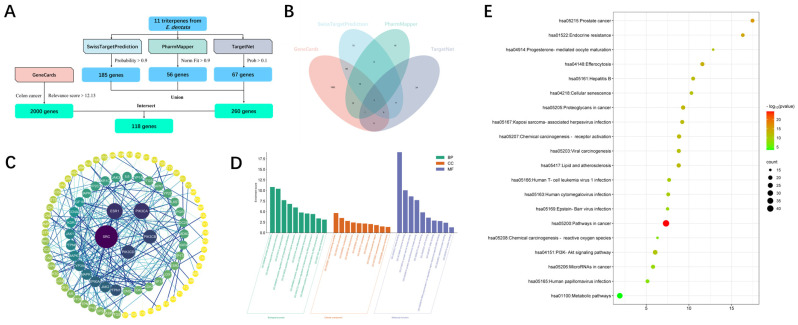
Target prediction and enrichment analysis results of triterpenoids from *E. dentata* and colon cancer. (**A**) The flowchart about the targets of triterpenoids and the target prediction of colon cancer. (**B**) The Venn diagram of triterpenoids—colon cancer targets. (**C**) The PPI network of the intersection target. (**D**) The GO enrichment analysis of the intersection targets. The green, orange, and purple columns respectively represent the intersecting targets related to Biological Process (BP), Cellular Components (CC), and Molecular Functions (MF). (**E**) The KEGG pathway enrichment analysis of common targets. The bubble size represents the number of intersecting targets enriched in a signaling pathway while the color of the bubble represents the p-value.

Given the exceptional cytotoxicity exhibited by compound **11** against the HT-29 cell line, we decided to conduct a bioinformatics analysis using network pharmacology to investigate the potential targets through which triterpenoids exert their cytotoxic effects. As shown in Figure 7A, within uploading the structures of the 11 triterpenoids, 185, 56, and 67 potential targets were collected from Swiss Target Prediction, PharmMapper, and TargetNet, respectively. Additionally, through GeneCards, we identified 2000 potential gene targets related to human colon cancer. By performing an intersection analysis using Venny 2.1.0, we identified 118 intersecting targets between the triterpenoid compounds and colon cancer (as shown in Figure 7B). Constructing a PPI network via STRING revealed prominent interactions among SRC, ESR, and PIK3CA (as shown in Figure 7C). Subsequently, we conducted GO and KEGG enrichment analyses. As shown in Figure 7D and 7E, the GO analysis results indicated that the intersecting targets are involved in processes such as protein phosphorylation, protein-containing complex assembly, and heme binding. According to the KEGG analysis, the intersecting targets are enriched in the PI3K-Akt signaling pathway.

### 2.4. Molecular Docking Analysis

Relevant studies have shown that one of the key pathways regulating the proliferation of HT-29 cells is the SRC signaling pathway, and the downstream PI3K/Akt phosphorylation signaling pathway of SRC is also a crucial pathway for the proliferation of cancer cells [41,42,43]. Based on the results of bioinformatics analysis, **11** is likely to exert its cytotoxic effects on HT-29 cells by modulating the SRC/PI3K/Akt signaling pathway. To further validate this hypothesis, we selected the SRC protein (PDB ID: 8JN8) and PIK3CA protein (PDB ID: 7R9V) for molecular docking with **11** to investigate their interactions.

**Figure 8:**
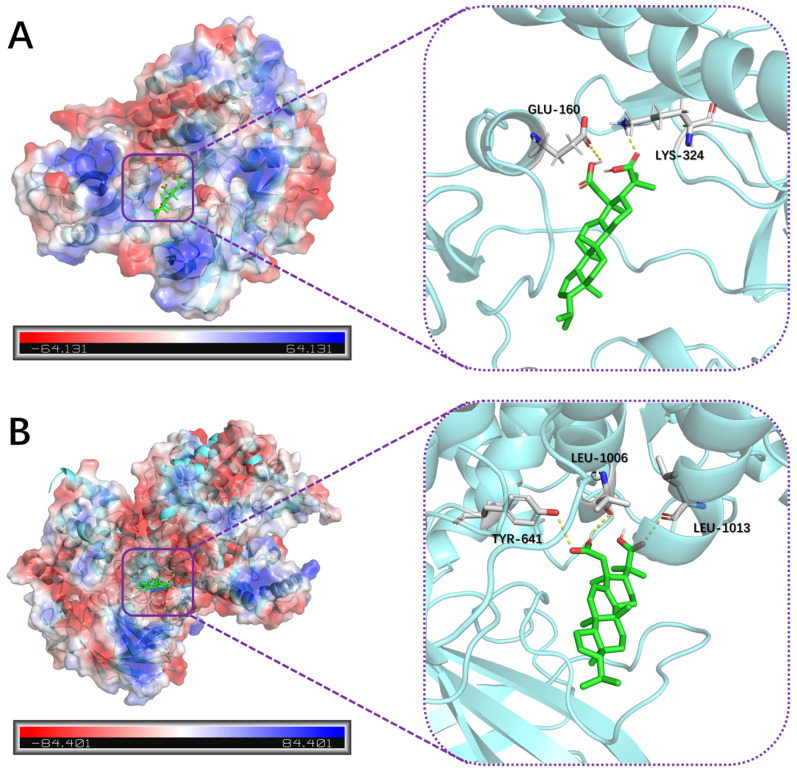
Molecular docking analysis of **11** with SRC protein (PDB ID: 8JN8, as shown in (**A**)) and PIK3CA protein (PDB ID: 7R9V, as shown in (**B**)).

The molecular docking results revealed that **11** successfully entered the active sites of SRC and PIK3CA proteins and formed hydrogen bonds with the target proteins. In the molecular docking with the SRC protein (as shown in Figure 8A), the hydroxyl hydrogen of 2-COOH of **11** formed a hydrogen bond with the carbonyl oxygen of GLU-160, and the carbonyl oxygen of 3-COOH formed a hydrogen bond with a hydrogen atom of LYS-324. In the molecular docking with the PIK3CA protein (as shown in Figure 8B), the carbonyl oxygen of 2-COOH of **11** formed a hydrogen bond with the phenolic hydroxyl hydrogen of TYR-641, the hydroxyl hydrogen of 2-COOH formed a hydrogen bond with the carbonyl oxygen of LEU-1006, and the carbonyl oxygen of 3-COOH formed a hydrogen bond with a hydrogen atom of LEU-1013.

These preliminary results suggest that compound **11** may exert its antitumor effects through the SRC/PI3K/Akt signaling pathway. However, more definitive results require further experimental validation, such as Western blotting.

## 3. Experiment

### 3.1. General Experimental Procedures

All 1D (^1^H and ^13^C NMR, DEPT-135) and 2D (HSQC, HMBC, COSY, and NOESY) NMR data were obtained from the Bruker Avance DRX600. All NMR data were analyzed with the MestReNova v14.0 software. The chemical shift values were presented in *δ* (ppm) based on the Dimethyl Sulfoxide-*d*_6_ (*δ*_C_ 39.5; *δ*_H_ 2.50, 3.33) resonance as references, whereas the coupling constants were expressed as J in Hz. All HR-ESI-MS data were obtained from the Agilent 1260–6224 liquid phase high-resolution time-of-flight mass spectrometer (Agilent Technologies, Inc., Beijing, China). All UV spectra were obtained with the Shimadzu UV-2550 spectrophotometer (Shimadzu (China) Co., Ltd, Shanghai, China). All IR spectra were obtained with the Nicolet 5700 FT-IR Spectrometer (Thermo Fisher Scientific Inc., Shanghai, China). All CD spectra were obtained with the JASCO J-1500 Circular Dichroism Spectrophotometer (JASCO China (Shanghai) Co., Ltd, Shanghai, China). All optical rotations were obtained with the INESA SGW-2 (Shanghai INESA Physico-Optical Instrument Co., Ltd, Shanghai, China). HPLC separations were performed with the Shimadzu LC-20AR (Shimadzu (China) Co., Ltd, Shanghai, China) equipped with a Fisher Wharton Xbridge C18 column (5 µm, 10 × 250 mm, Waters (Shanghai) Co., Ltd, Shanghai, China).

All HPLC-grade solvents in HPLC separations were from Honeywell (China) Co., Ltd (Shanghai, China). Column chromatography (CC) was performed with silica gel (80–100 mesh, 200–300 mesh, Qingdao Haiyang Chemical Co., Ltd., Qingdao, China), MCI GEL CHP20P (Mitsubishi Chemical Corporation, Tokyo, Japan), D101-macroporous absorption resin (Shanghai Yuanye Bio-Technology Co., Ltd., Qingdao, China), and Sephadex LH-20 (Sigma-Aldrich (Shanghai) Trading Co., Ltd., Qingdao, China). Thin-layer chromatography (TLC) was performed with precoated silica gel GF254 plates (Qingdao Haiyang Chemical Co., Ltd., Qingdao, China). The TLC analysis was performed with 10% sulfuric acid-ethanol as the chromogenic agent. All analytical grade solvents in CC and TLC were from Sinopharm Chemical Reagent Co., Ltd. (Shanghai, China). All deuterated solvents (Dimethyl Sulfoxide-*d*_6_, 99.0%) were purchased from Shanghai Titan Scientific Co., Ltd., Shanghai, China.

### 3.2. Plant Material

The whole-plant sample of *E. dentata* was collected in November, 2020, from Yunnan Province, People’s Republic of China. The sample was identified and classified by Anrui Lou, the senior engineer from Kunming Plant Biotechnology Co., Ltd. (Kunming, China), with reference to specimens in the Herbarium of the School of Life Sciences at Beijing Normal University. The sample of *E. dentata* was air-dried naturally and stored under cool and dry conditions. A voucher specimen (accession number: CLDJ-2020-11-01) was deposited at the Natural Products Chemistry Laboratory, College of Chemistry and Chemical Engineering, Southeast University, Nanjing, China.

### 3.3. Extraction and Isolation

The entire dried plant of *E. dentata* (11.25 kg) was subjected to thermal reflux extraction with 85% EtOH (6 h × 3 × 40 L) at 80 °C, which yielded 1.47 kg of ethanol extract. The crude extract of *E. dentata* was then dispersed in warm deionized water (3 L) and sequentially extracted three times each with petroleum ether (3 × 5 L), ethyl acetate (3 × 5 L), and n-butanol (3 × 5 L). After concentration under reduced pressure, the PE-fraction (254.4 g), EA-fraction (148.3 g), and nB-fraction (157.8 g) extracts were obtained.

PE-fraction (254.4 g) was separated on the silica gel column (200–300 mesh, PE/EA, 20:1 to 0:1, *v*/*v*) to give PE-fraction1 to PE-fraction9. PE-fraction3 was separated on the MCI GEL CHP20P (MeOH/H_2_O, 7:3 to 1:0, *v*/*v*) to give PE-fraction3-1 to PE-fraction3-5. PE-fraction3-1 (2.67 g) was separated with recrystallizing to obtain **12** (164.2 mg). PE-fraction4 was separated on the silica gel column (200–300 mesh, DCM/MeOH, 1:0 to 0:1, *v*/*v*) to give PE-fraction4-1 to PE-fraction4-9. PE-fraction4-2 (59.3 mg) was separated with preparative TLC (PE/AC, 3:1, *v*/*v*) to obtain **1** (3.4 mg). PE-fraction4-7 was separated on the silica gel column (200–300 mesh, PE/AC, 1:0 to 0:1, *v*/*v*) to give PE-fraction4-7-1 to PE-fraction4-7-18. PE-fraction4-7-3 (0.93 g) was separated on the silica gel column (200–300 mesh, DCM/MeOH, 1:0 to 0:1, *v*/*v*) to obtain **13** (17.0 mg). PE-fraction4-7-5 (1.15 g) was separated on the silica gel column (200–300 mesh, DCM/MeOH, 20:1 to 0:1, *v*/*v*) to obtain **2** (34.7 mg), **7** (11.3 mg), and **8** (5.9 mg). PE-fraction4-7-8 (355.3 mg) was separated on the silica gel column (200–300 mesh, DCM/MeOH, 1:0 to 0:1, *v*/*v*) to obtain **3** (1.7 mg), **4** (3.3 mg), **16** (9.9 mg), and **10** (12.6 mg). Based on the TLC analysis, PE-fraction5 and PE-fraction6 were combined for further separation. PE-fraction5/6 (12.71 g) was separated on the D101-macroporous absorption resin (EtOH/H_2_O, 7:3 to 1:0, *v*/*v*) to give PE-fraction5-1 to PE-fraction5-5. PE-fraction5-2 (2.94 g) was separated on the silica gel column (200–300 mesh, PE/EA, 30:1 to 0:1, *v*/*v*) to give PE-fraction5-2-1 to PE-fraction5-2-13 together with obtaining **5** (3.1 mg). PE-fraction5-2-13 (0.23 g) was separated on semipreparative HPLC (85% CH_3_CN in H_2_O, 3 mL/min) to obtain **19** (t*_R_* = 41.3 min, 49.0 mg). PE-fraction5-4 (1.1 g) was separated on the silica gel column (200–300 mesh, PE/AC, 30:1 to 0:1, *v*/*v*) to obtain **6** (10.8 mg) and **9** (4.3 mg). Based on the TLC analysis, PE-fraction7 and PE-fraction8 were combined for further separation. PE-fraction7/8 (23.01 g) was separated on the D101-macroporous absorption resin (EtOH/H_2_O, 7:3 to 1:0, *v*/*v*) to give PE-fraction7-1 to PE-fraction7-4. PE-fraction7-3 (5.8 g) was separated on the Sephadex LH-20 (MeOH) to give PE-fraction7-3-1 to PE-fraction7-3-7. PE-fraction7-3-5 (2.7 g) was separated on the silica gel column (200–300 mesh, PE/EA, 10:1 to 0:1, *v*/*v*) to give PE-fraction7-3-5-1 to PE-fraction7-3-5-19 together with obtaining **14** (146.7 mg), **15** (50.7 mg), and **20** (9.0 mg). PE-fraction7-3-5-10 (0.4 g) was separated on semipreparative HPLC (98% CH_3_CN in H_2_O, 3 mL/min) to obtain **17** (t*_R_* = 26.1 min, 21.3 mg) and **18** (t*_R_* = 29.8 min, 73.8 mg). PE-fraction7-3-6 (95 mg) was purified on semipreparative HPLC (95% CH_3_CN in H_2_O, 3 mL/min) to obtain **11** (t*_R_* = 29.5 min, 6.7 mg).

### 3.4. Compounds Characterization

Dentatacid A (**11**): white powder (chloroform/methanol). [α${]}_{D}^{20}$ +86 (c 0.005, MeOH). UV (MeOH) *λ*_max_ (log ε) 195 (7.14), 198 (7.02) nm. ECD (MeOH) *λ* (Δ*ε*) 200 (+5.78), 222 (+16.478), 258 (−0.18) nm; IR (KBr) *ν*_max_ 3443, 1592,1384, 1351, 618 cm^−1^; ^1^H and ^13^C NMR data (Dimethyl Sulfoxide-*d*_6_), as shown in Table 1. (+)-HR-ESI-MS m/z 495.3441 [M + Na]^+^ (calcd for C_30_H_48_NaO_4_^+^, 495.3446). All data of dentatacid A’s characterization are shown in Figures S1–S13.

### 3.5. ECD Calculations

Conformational analysis was performed with Monte Carlo searching in the MMFF94 molecular mechanics force field in Spartan ‘14 V1.1.4 [44]. The conformers with the Boltzmann distribution greater than 0.05 were considered for further DFT calculations.

Subsequently, the conformers were optimized further using DFT at the B3LYP/6-31G(d) level in the methanol with the GAUSSIAN 16 [45]. The theoretically calculated ECD spectra of **11** were established using the time-dependent density functional theory (TDDFT) method at the B3LYP/6-311++G(2d,2p) level in methanol, and further Boltzmann averaged to compare with experimentally obtained ECD spectra by SpecDis v1.71 [46,47]. The sigma/gamma ratio for processing the calculated ECD was 0.3 eV. All data of ECD calculations are shown in Figure S14 and Tables S1 and S2.

### 3.6. Methods of Cytotoxicity Assay

#### 3.6.1. Materials

Dulbecco’s modified Eagle’s medium (DMEM), fetal bovine serum (FBS), and antibiotics (penicillin/streptomycin) were purchased from Jiangsu KeyGEN Bio TECH Corp., Ltd. (Nanjing, China). Cell Counting Kit-8 (CCK-8) was purchased from Beyotime Biotech, Inc. (Shanghai, China). 143B, A549, HepG2, and HT-29 were purchased from the National Collection of Authenticated Cell Cultures (Shanghai, China).

#### 3.6.2. Cell Culture

All 143B, A549, HepG2, and HT-29 were cultured with DMEM containing 10% FBS and 1% antibiotics (penicillin/streptomycin) at 37 °C incubator in a humidified atmosphere of 5% CO_2_.

When cells reach 80–90% confluency within a single field of view, the cells in the logarithmic growth phase will undergo subculture. Digestion of the cells is performed using 0.25% trypsin with EDTA, and the digestion process is terminated using a complete DMEM medium. The subcultured cells will be maintained at a density of 1–2 × 10^6^ cells per milliliter of medium.

#### 3.6.3. Cell Viability

Cell viability of 143B, A549, HepG2, and HT-29 was measured by the CCK-8 assay. Cells were seeded in 96-well plates at a density of 5 × 10^6^ cells/well for 24 h and subsequently treated with the tested compounds for another 24 h. After exposure, the medium was replaced with 100 µL complete DMEM medium containing 10% CCK-8 and maintained the culture for 4 h. Then the absorbance was measured at 450 nm using the Thermo Scientific Multiskan GO (Thermo Fisher Scientific, Waltham, MA, USA). GraphPad Prism 10 was used to conduct all statistical analyses.

### 3.7. Methods of Bioinformatics Analysis

#### 3.7.1. Target Prediction of Triterpenoids from *E. dentata*

The targets of triterpenoids of *E. dentata* and colon cancer were collected from Swiss Target Prediction (http://www.swisstargetprediction.ch/, accessed on 1 August 2024) [48], PharmMapper (https://lilab-ecust.cn/pharmmapper/index.html, accessed on 1 August 2024) [49] and TargetNet (http://targetnet.scbdd.com/, accessed on 1 August 2024) [50]. The targets from the Swiss Target Prediction were with a probability > 0.1. The targets from PharmMapper were with the Norm Fit > 0.9. The targets from TargetNet were with the Prob > 0.1.

#### 3.7.2. Screening of Potential Targets in Colon Cancer

The potential targets of colon cancer were collected from GeneCards (https://www.genecards.org/, accessed on 1 August 2024) with colon cancer as the keyword [51]. The targets from GeneCards had a relevance score of > 12.13.

#### 3.7.3. Construction of the Target PPI Network between Triterpenoids from *E. dentata* and Colon Cancer

Venny 2.1.0 (https://bioinfogp.cnb.csic.es/tools/venny/, accessed on 1 August 2024) was employed to intersect the targets of triterpenoids from *E. dentata* and colon cancer. The intersection targets were submitted to STRING11.0 (https://cn.string-db.org/, accessed on 1 August 2024) [52] to construct the Protein-Protein Interaction (PPI) network model. The PPI network model was constructed in multiple proteins of STRING11.0 with *Homo sapiens* of the Organisms and with the medium confidence (0.400) of the minimum required interaction score. CytoScape3.10.1 (Free Software Foundation, Inc., Boston, MA, USA) was employed to visualize the PPI network. The PPI network was analyzed with Degree, Closeness, and Betweenness generated from the app CentiScaPe 2.2 in CytoScape3.10.1.

#### 3.7.4. Enrichment Analysis of the Intersection Targets

The targets of intersection targets were submitted to the Database for Annotation, Visualization, and Integrated Discovery (DAVID) Bioinformatics (https://david.ncifcrf.gov/, accessed on 1 August 2024), and the main metabolic pathways were analyzed and enrichment analysis was performed [53]. The result of the enrichment analysis was submitted to WeiShengXin (https://www.bioinformatics.com.cn/, accessed on 1 August 2024) to visualize the GO enrichment analysis and KEGG pathway enrichment analysis.

### 3.8. Molecular Docking

The lowest energy conformations of the compound were from the ECD result with the highest Boltzmann distribution, further optimizing with MM2—Minimize Energy in Chem3D 20.0 (PerkinElmer, Inc., Waltham, MA, USA). The crystal structure of SRC protein (PDB code: 8JN8) and PI3K protein (PDB code: 7R9V) was obtained from the protein data bank (PDB, http://www.wwpdb.org, accessed on 1 August 2024). The structure of the SRC and PI3K proteins was optimized using Open-Source PyMOL (Schrödinger, LLC, New York, NY, USA), including removal of water molecules, hydrogenation, and energy minimization. The molecular docking between the compounds and two proteins was performed using AutoDockTools-1.5.7 and AutoDock vina [54]. The visualization of the molecular docking results was also performed using Open-Source PyMOL.

## 4. Conclusions

In summary, we isolated 11 triterpenoids, 7 sterols, a diterpenoid, and a sesquiterpenoid from the invasive plant *E. dentata*, including a structurally unprecedented 2, 3-seco-arbor-2, 3-dioic triterpenoid. Through preliminary investigations involving cytotoxicity assays, bioinformatics analysis, and molecular docking, we have made significant progress in exploring the antitumor activity of these triterpenoids. Among them, compound 11 showed notably more compelling cytotoxicity with an IC50 value of 2.64 ± 0.78 µM, surpassing HCPT’s IC50 value of 2.70 ± 0.16 µM for HT-29. These findings greatly enhance the understanding of the secondary metabolites and biological activities of *E. dentata* and offer new insights into the resource utilization and management of this invasive plant from the perspective of natural product discovery.

## Figures and Tables

**Table 1:** ^1^H (600 MHz) and ^13^C (150 MHz) NMR data of **11** in Dimethyl Sulfoxide-*d*_6_.

No.	*δ*_C_	*δ*_C_, Type	*δ*_H_, (mult, *J*)
1	42.0	CH_2_	2.67 (d, *J* = 18.0 Hz), 2.56 (d, *J* = 18.0 Hz)
2	172.4	C	-
3	180.6	C	-
4	45.6	C	-
5	46.7	CH	2.52 (dd, *J* = 11.9, 3.2 Hz)
6	23.6	CH_2_	_α_ 1.65 ^a^ (m), _β_ 1.50 ^a^ (m)
7	24.5	CH_2_	_β_ 1.68 ^a^ (m), _α_ 1.17 ^a^ (m)
8	41.8	CH	1.91 (d, *J* = 13.1 Hz)
9	145.9	C	-
10	43.4	C	-
11	114.5	CH	5.23 (d, *J* = 5.7 Hz)
12	36.2	CH_2_	_α_ 1.59 (d, *J* = 2.9 Hz), _β_ 1.39 ^a^ (m)
13	35.9	C	-
14	37.9	C	-
15	29.0	CH_2_	_β_ 1.21 (s), _α_ 1.27 ^a^ (m)
16	35.5	CH_2_	_α_ 1.34 (dd, *J* = 13.3, 2.9 Hz), _β_ 1.59 (dd, *J* = 13.3, 2.9 Hz)
17	42.4	C	-
18	51.5	CH	1.52 ^a^ (m)
19	19.7	CH_2_	1.28 ^a^ (m)
20	27.8	CH_2_	1.15 ^a^ (m), 1.76 (m)
21	59.0	CH	0.94 (q, *J* = 9.4 Hz)
22	30.3	CH	1.39 ^a^ (m)
23	27.3	CH_3_	1.09 ^a^ (s)
24	23.5	CH_3_	1.10 (s)
25	25.3	CH_3_	1.09 ^a^ (s)
26	16.5	CH_3_	0.74 (s)
27	15.3	CH_3_	0.71 (s)
28	13.8	CH_3_	0.70 (s)
29	22.9	CH_3_	0.79 (d, *J* = 6.5 Hz)
30	22.0	CH_3_	0.85 (d, *J* = 6.5 Hz)

^a^ Multiplicity not determined due to overlapping signals.

**Table 2:** Cytotoxic assay of compounds **1**–**11** with IC_50_ values (µM).

Compounds	A549 (μM) ^a^	HepG2 (μM) ^a^	HT-29 (μM) ^a^	143B (μM) ^a^
**1**	22.79 ± 2.86	>50	25.10 ± 1.87	19.98 ± 3.16
**2**	22.69 ± 1.46	24.32 ± 1.40	15.66 ± 0.38	>50
**3**	>50	12.29 ± 0.11	11.87 ± 2.73	19.11 ± 5.53
**4**	4.74 ± 2.61	10.10 ± 1.04	8.92 ± 2.82	26.34 ± 1.06
**5**	31.56 ± 6.57	26.68 ± 4.00	5.94 ± 2.13	>50
**6**	>50	24.82 ± 1.13	22.77 ± 3.66	26.98 ± 0.65
**7**	10.04 ± 2.22	>50	18.90 ± 1.56	>50
**8**	9.63 ± 2.25	>50	21.48 ± 4.15	19.27 ± 1.95
**9**	29.68 ± 3.69	23.68 ± 1.72	15.07 ± 13.56	17.01 ± 2.73
**10**	>50	31.52 ± 4.42	13.01 ± 1.14	>50
**11**	4.85 ± 1.14	10.65 ± 2.17	2.64 ± 0.78	18.82 ± 0.60
**HCPT ^b^**	2.89 ± 1.47	3.19 ± 0.46	2.70 ± 0.16	2.56 ± 1.41

^a^ The values presented are the mean ± SD of triplicate experiments. ^b^ The positive control. The bold means different compounds.

## Data Availability

Data are contained within the article and Supplementary Materials.

## Supplementary Materials

The following supporting information can be downloaded at: https://www.mdpi.com/article/10.3390/plants13172533/s1. Figure S1: The structure (A), the key HMBC and 1H–1H COSY correlations (B) of 11; Figure S2. Key 1H-1H NOESY correlations correlations of 11; Figure S3. 1H NMR spectrum of 11 in Dimethyl Sulfoxide-d6; Figure S4. 13C NMR spectrum of 11 in Dimethyl Sulfoxide-d6; Figure S5. DEPT-135 spectrum of 11 in Dimethyl Sulfoxide-d6; Figure S6. HSQC spectrum of 11 in Dimethyl Sulfoxide-*d_6_*; Figure S7. HMBC spectrum of 11 in Dimethyl Sulfoxide-*d_6_*; Figure S8. 1H-1H COSY spectrum of 11 in Dimethyl Sulfoxide-*d_6_*; Figure S9. 1H-1H NOESY spectrum (A) and the partial enlarged detail (B) of 11 in Dimethyl Sulfoxide-*d_6_*; Figure S10. (+)-HR-ESI-MS spectrum (A) and the partial enlarged detail (B) of 11; Figure S11. UV spectrum of 11; Figure S12. Calculated and experimental ECD spectra for 11; Figure S13. IR spectrum of 11; Figure S14. Conformers of isomer 11; Table S1. Important thermodynamic parameters and conformational analysis of 11 at the B3LYP/6-311++G(2d,2p) level with CPCM solvent model in methanol; Table S2. Cartesian coordinates for the reoptimized conformers of 11 at the B3LYP/6-311++G(2d,2p) level with CPCM solvent model in methanol; The NMR data for known compounds.
