# Supplementary material for: Dentatacid A: An Unprecedented 2, 3-Seco-arbor-2, 3-dioic Triterpenoid from the Invasive Plant Euphorbia dentata, with Cytotoxicity Effect on Colon Cancer
Source: Plants (Basel). 2024 Sep 9;13(17):2533. doi: 10.3390/plants13172533 (PMC11397642; doi:10.3390/plants13172533)
Supplement: Supplementary file 1 [file plants-13-02533-s001.zip › plants-3190216-supplementary.pdf]

Supplementary data

Dentatacid A: an unprecedented 2,3-*seco*-arbor-2,3-dioic triterpenoid from the invasive plant *Euphorbia dentata*, with cytotoxicity effect on colon cancer

Chen-Sen Xu [a], Yuan-Ling Shao [a, b], Qing Li [a], Yu Zhang [a], Hong-Wei Wu [a], Hao-Lin Yu [a], Yun-Yun Su [a], Jing Zhang [a], Chao Wang [a], Zhi-Xin Liao [a] *

[a] *Department of Pharmaceutical Engineering, School of Chemistry and Chemical Engineering and Jiangsu Province Hi-Tech Key Laboratory for Biomedical Research, Southeast University, Nanjing, 211189, PR China;*

[b] *Changshu Institute for Products Quality Supervision and Inspection, Changshu Measurement and Testing Center, Suzhou, 215500, PR China.*

**Corresponding author.*

E-mail address: zxliao@seu.edu.cn

Contents of *Supplementary data*

Figure S1. The structure (A), the key HMBC and 1H–1H COSY correlations (B) of **11**.................3

Figure S2. Key ^{1}H-^{1}H NOESY correlations correlations of **11** 4

Figure S3. ^{1}H NMR spectrum of **11** in Dimethyl Sulfoxide-d_6..4

Figure S4. ^{13}C NMR spectrum of **11** in Dimethyl Sulfoxide-d_6 5

Figure S5. DEPT-135 spectrum of **11** in Dimethyl Sulfoxide-d_6..5

Figure S6. HSQC spectrum of **11** in Dimethyl Sulfoxide-d_6 6

Figure S7. HMBC spectrum of **11** in Dimethyl Sulfoxide-d_6 6

Figure S8. ^{1}H-^{1}H COSY spectrum of **11** in Dimethyl Sulfoxide-d_6 7

Figure S9. ^{1}H-^{1}H NOESY spectrum (A) and the partial enlarged detail (B) of **11** in Dimethyl Sulfoxide-d_6 8

Figure S10. (+)-HR-ESI-MS spectrum (A) and the partial enlarged detail (B) of 11. 9

Figure S11. UV spectrum of **11**. 10

Figure S12. Calculated and experimental ECD spectra for **11**...11

Figure S13. IR spectrum of **11**. 11

Figure S14. Conformers of isomer **11**...12

Table S1. Important thermodynamic parameters and conformational analysis of **11** at the B3LYP/6-311++G(2d,2p) level with CPCM solvent model in methanol......................................12

Table S2. Cartesian coordinates for the reoptimized conformers of **11** at the B3LYP/6-311++G(2d,2p) level with CPCM solvent model in methanol. 12

The NMR data for known compounds...27

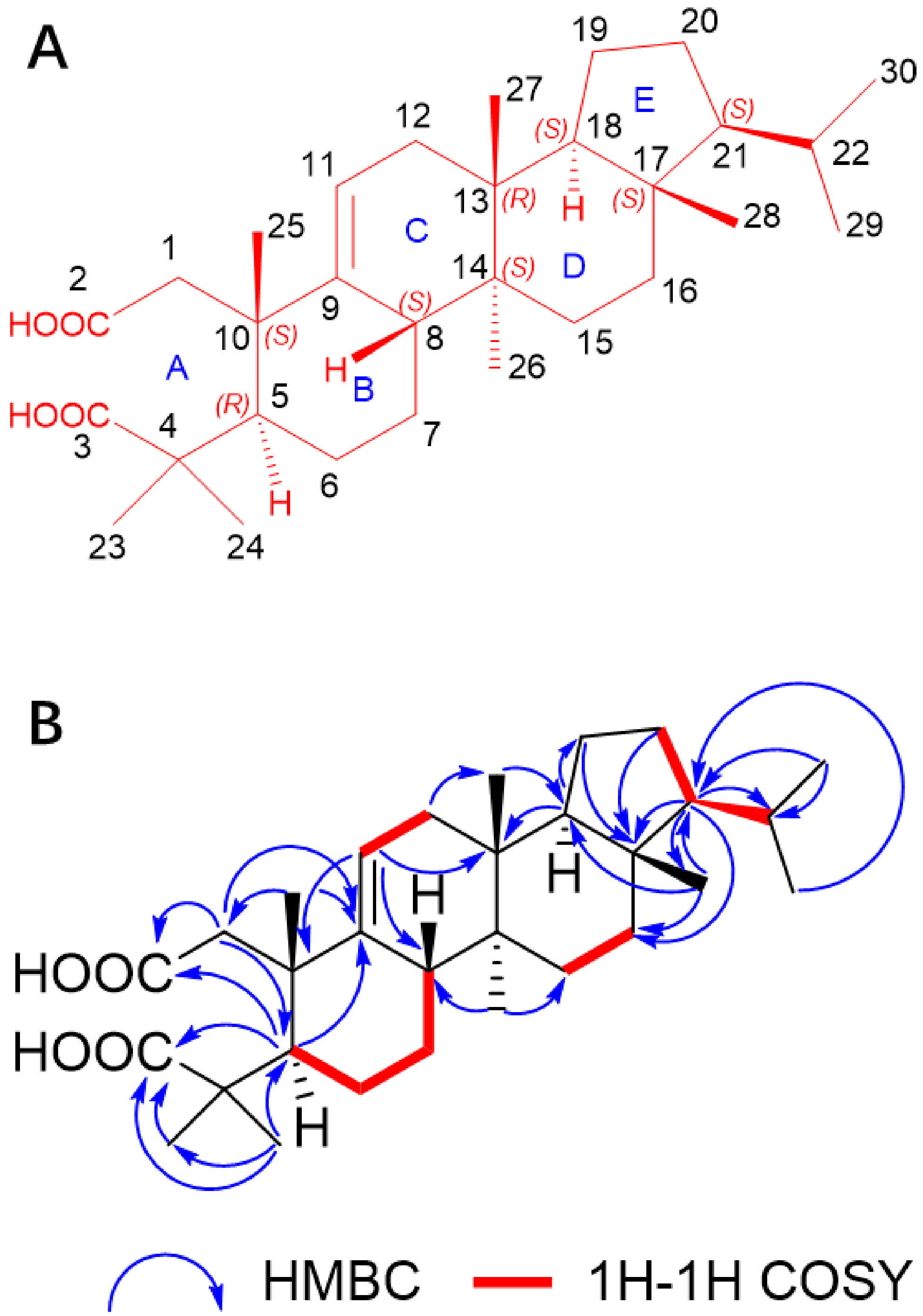

Figure S1. The structure (A), the key HMBC and 1H–1H COSY correlations (B) of **11**.

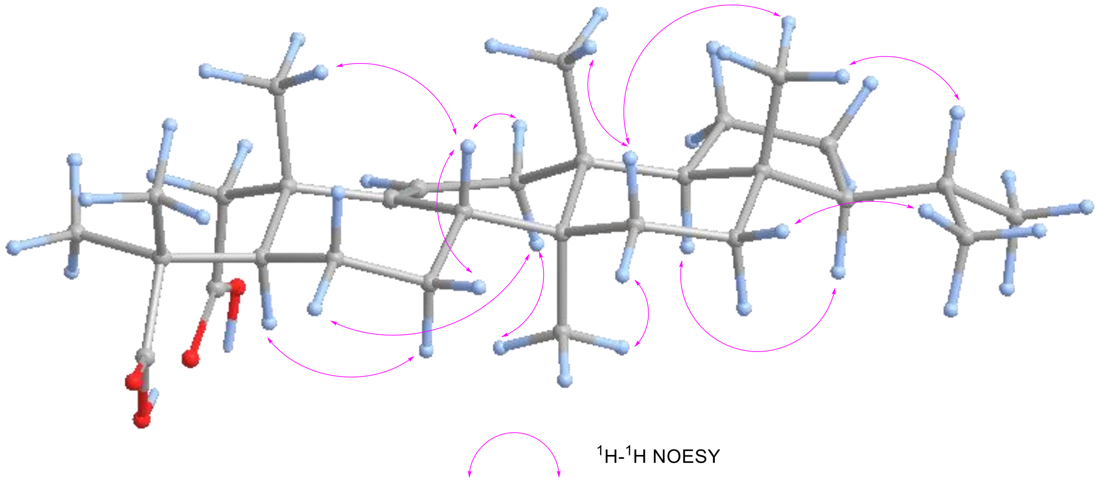

Figure S2. Key ^{1}H-^{1}H NOESY correlations correlations of **11**

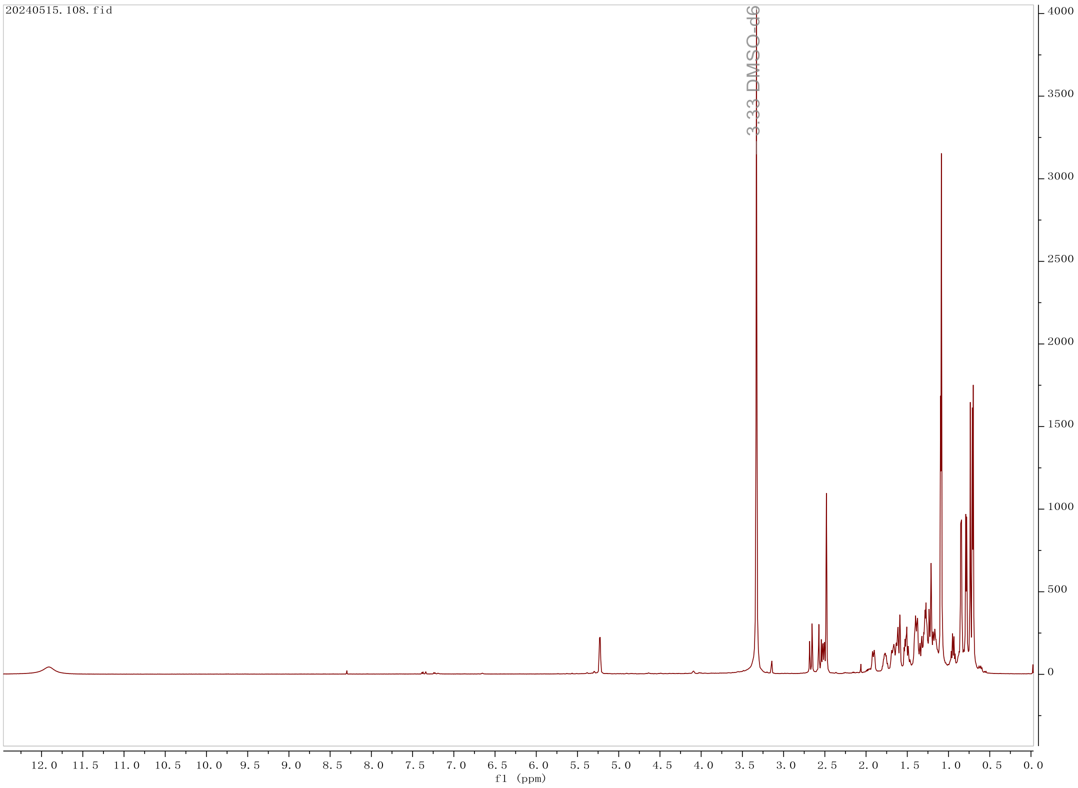

Figure S3. ^{1}H NMR spectrum of **11** in Dimethyl Sulfoxide-d_6

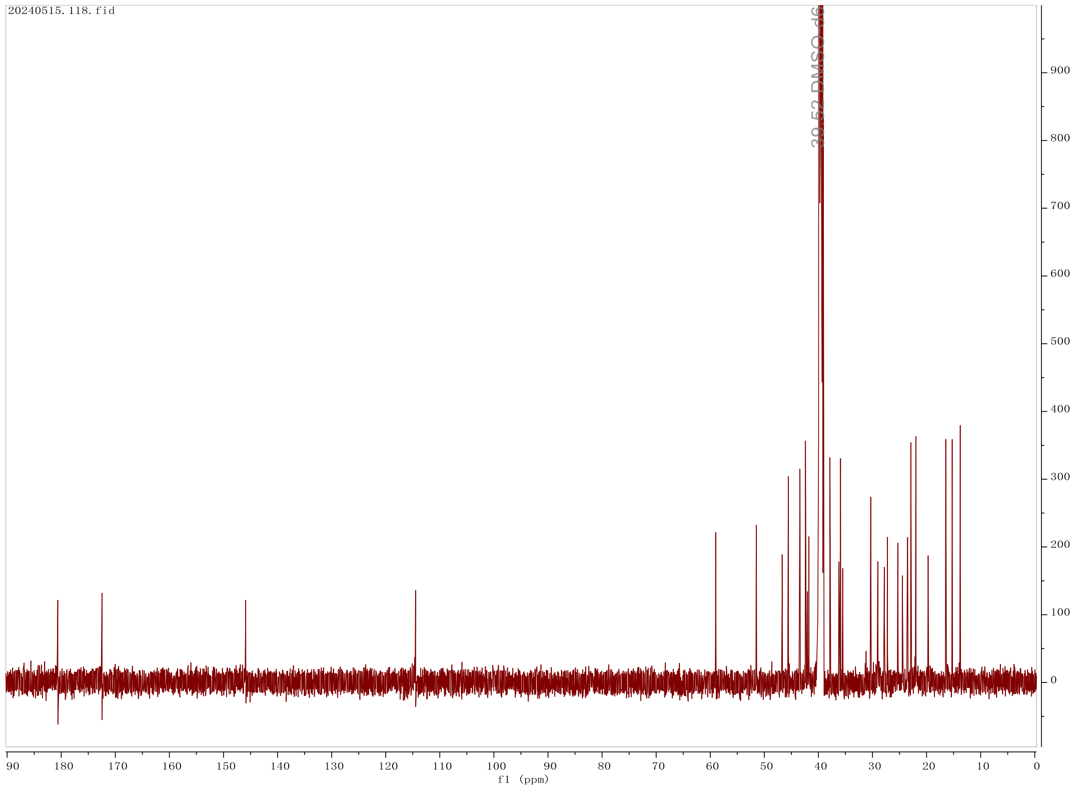

Figure S4. ^{13}C NMR spectrum of **11** in Dimethyl Sulfoxide-d_6

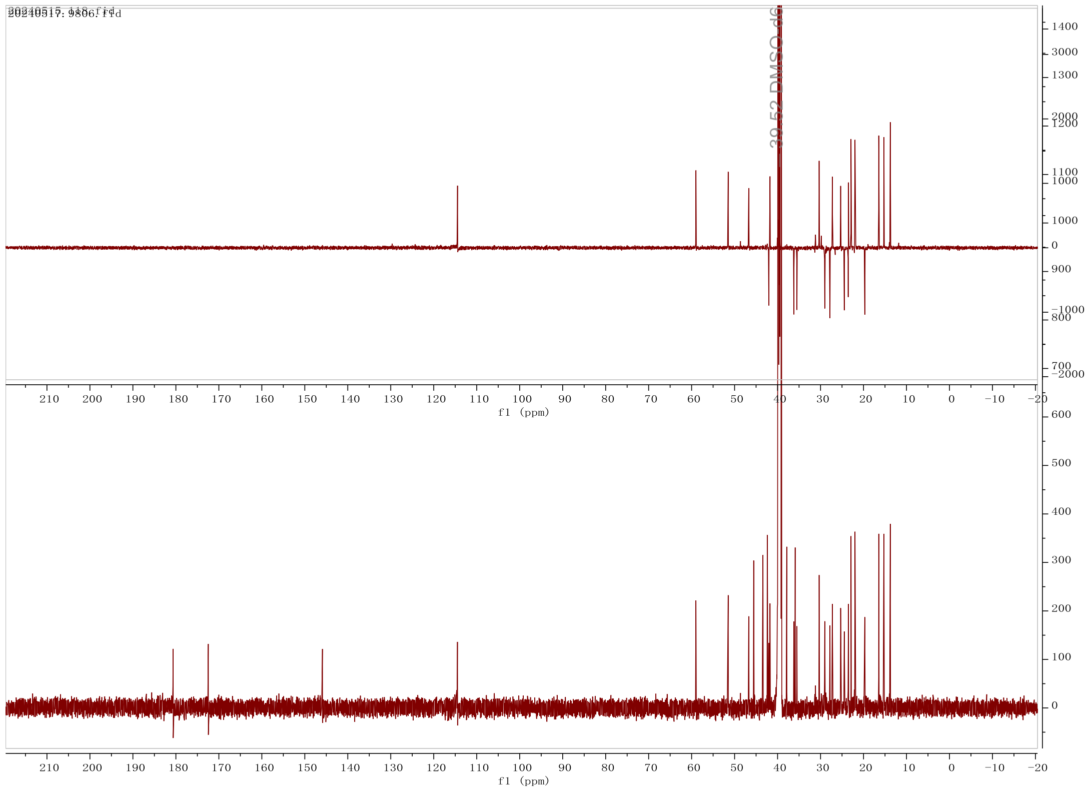

Figure S5. DEPT-135 spectrum of **11** in Dimethyl Sulfoxide-d_6

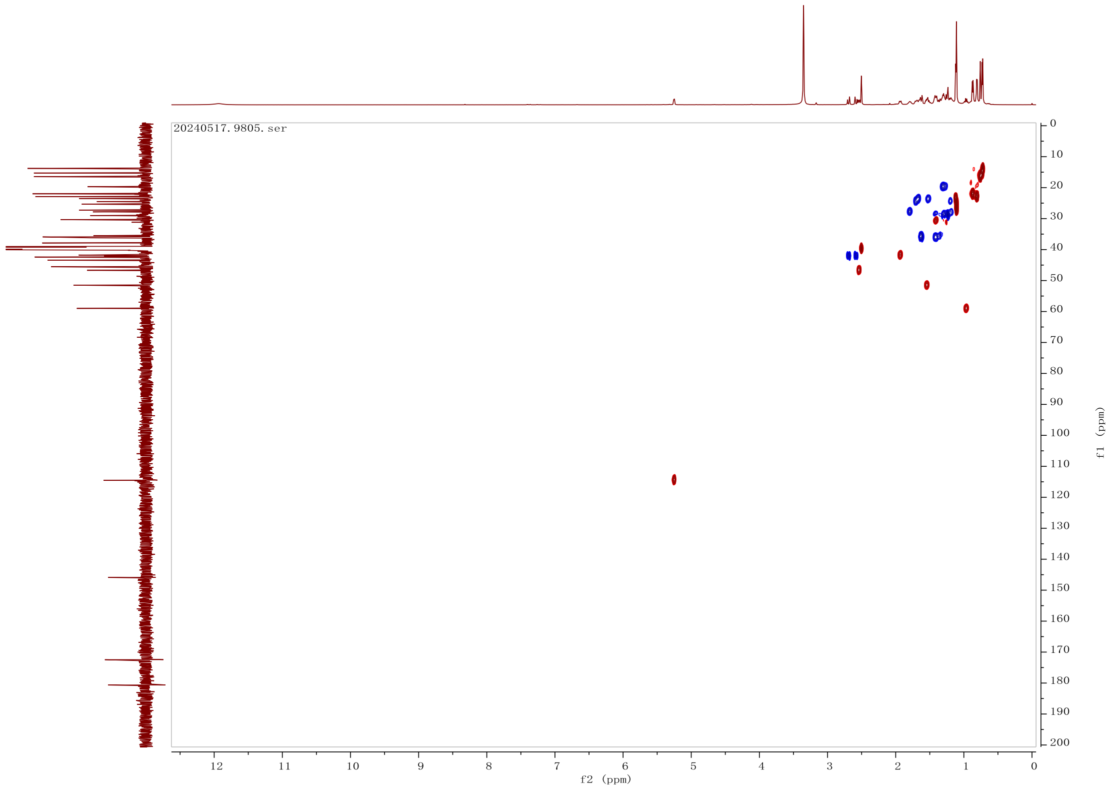

Figure S6. HSQC spectrum of **11** in Dimethyl Sulfoxide-d_6

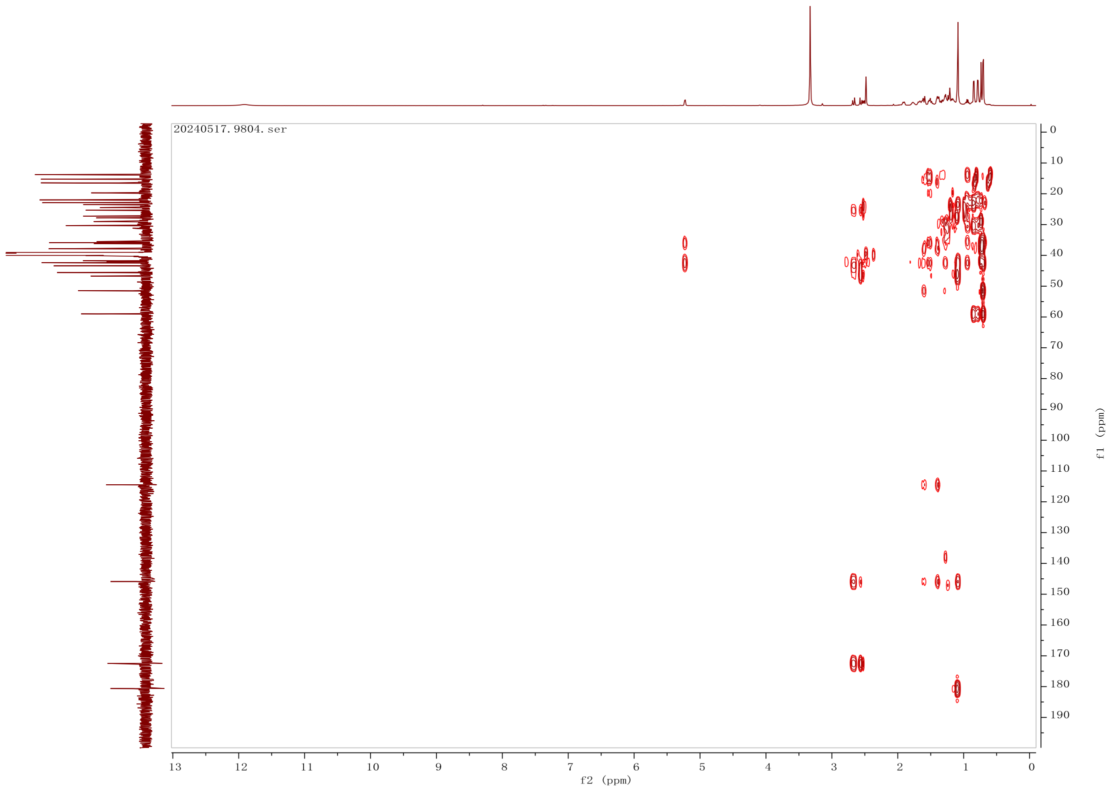

Figure S7. HMBC spectrum of **11** in Dimethyl Sulfoxide-d_6

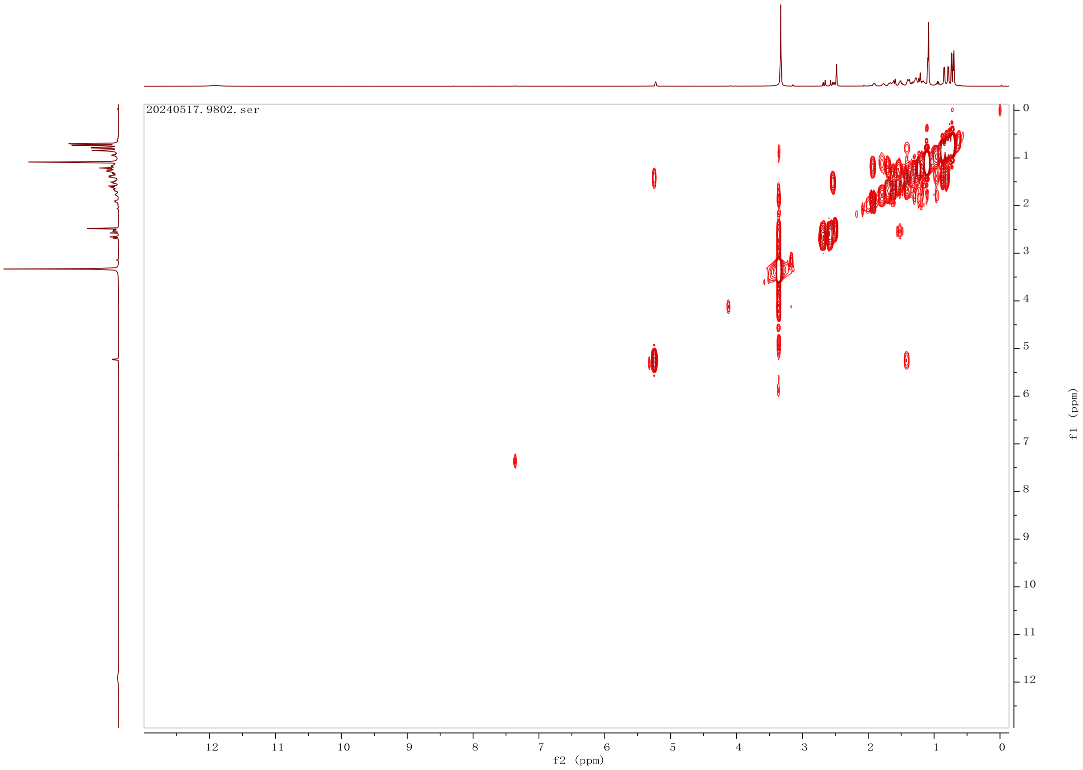

Figure S8. ^{1}H-^{1}H COSY spectrum of **11** in Dimethyl Sulfoxide-d_6

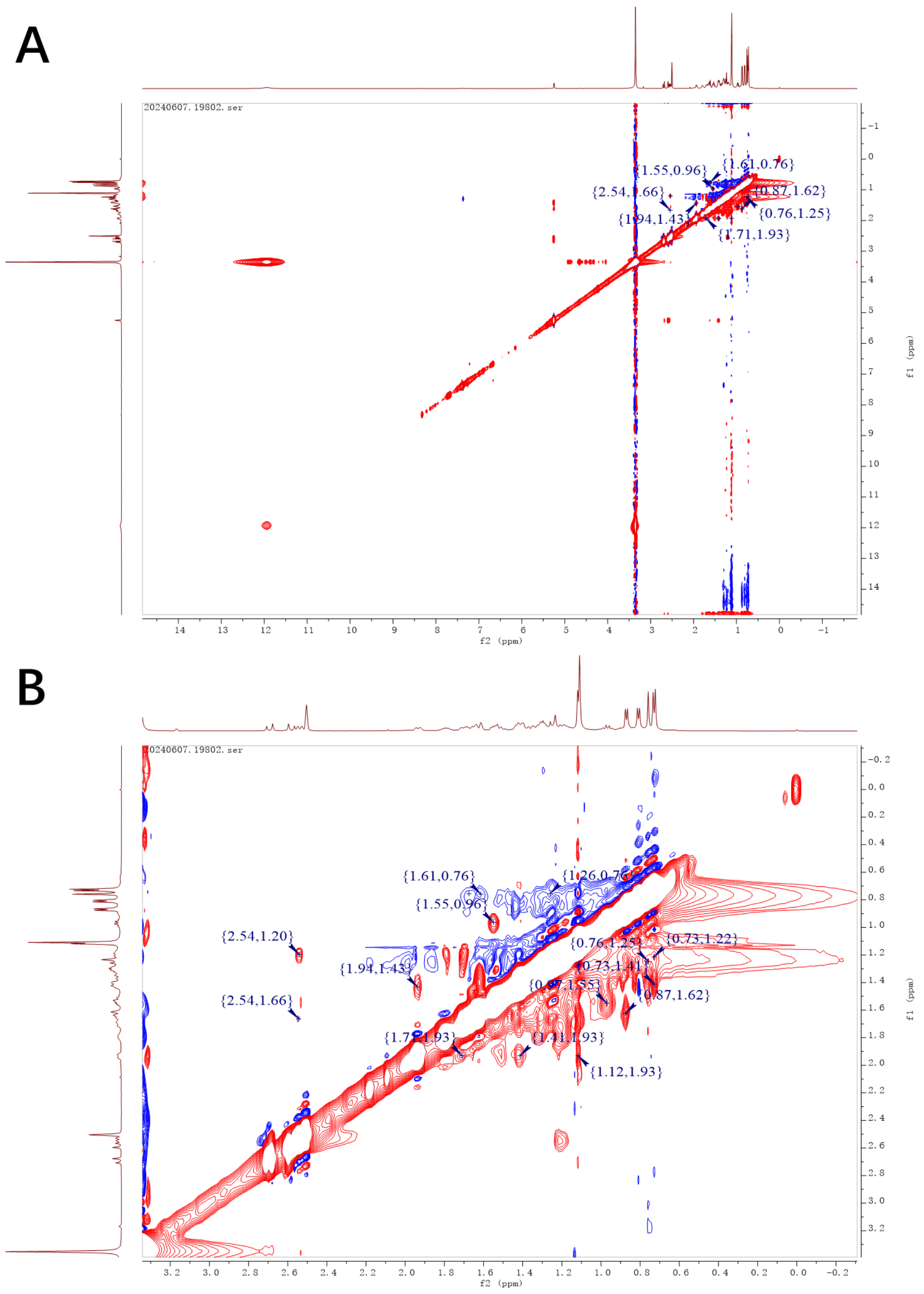

Figure S9. ^{1}H-^{1}H NOESY spectrum (A) and the partial enlarged detail (B) of **11** in Dimethyl Sulfoxide-d_6

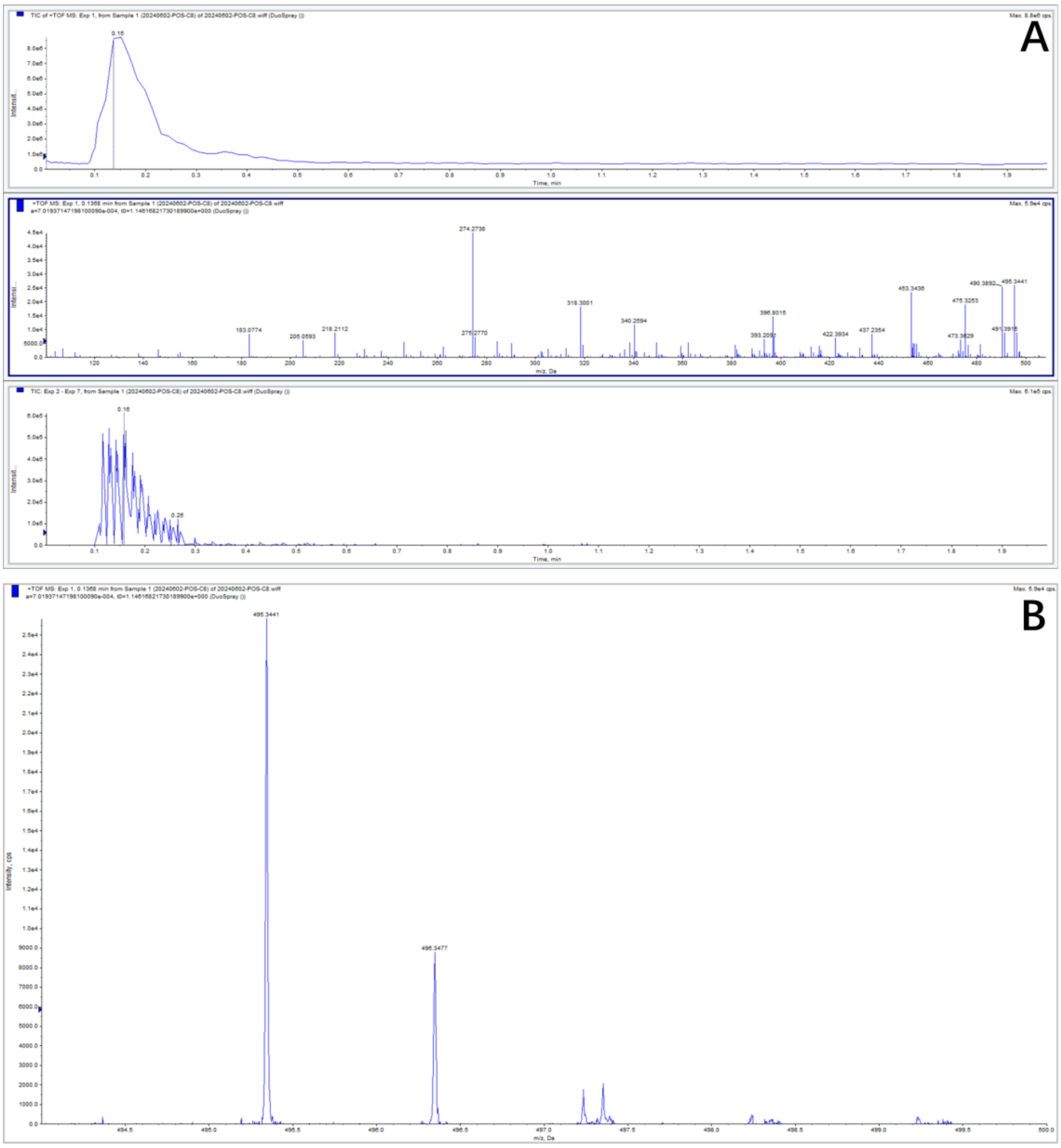

Figure S10. (+)-HR-ESI-MS spectrum (A) and the partial enlarged detail (B) of 11.

Spectral Peak Detection Report

光谱峰值检测报告

2024/06/14 12:08:43

数据集: CL08_120333 - RawData

Dataset

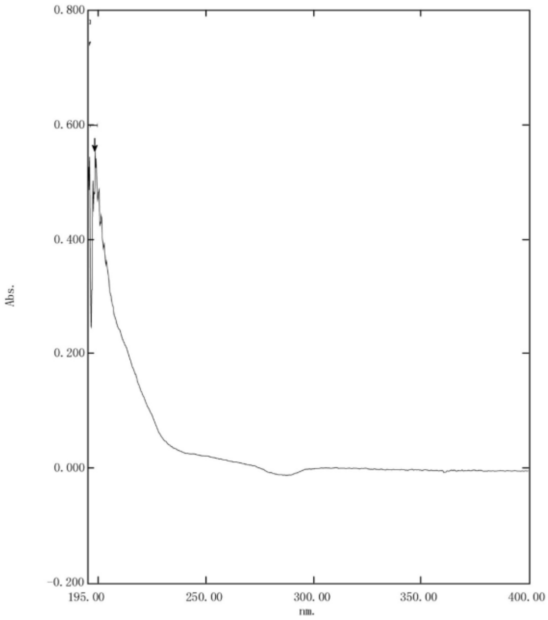

λ (nm)

[测定属性]
波长范围(nm): 190. 00 到 400. 00
扫描速度: 高速
采样间隔: 0. 2
自动采样间隔: 停用
扫描模式: 自动

[仪器属性]
仪器类型: UV-2500PC 系列
测定方式: 吸收值
狭缝宽: 2. 0 nm
光源转换波长: 360. 0 nm
S/R 转换: 标准

[附件属性]
附件: 无

[数据处理参数]
阈值: 0. 0010000
点: 4
内插: 启用
间隔: 0. 000100
平均: 停用

[Measured Properties]
Wavelength Range (nm): 190.00 to 400.00
Scanning Speed: High
Sampling Interval: 0.2
Automatic Sampling Interval: Disabled
Scanning Mode: Automatic

[Instrument Properties]
Instrument Type: UV-2500PC Series
Measurement Mode: Absorbance
Slit Width: 2.0 nm
Source Conversion Wavelength: 360.0 nm
S/R Conversion: Standard

[Attachment Properties]
Attachment: None

[Data Processing Parameters]
Threshold: 0.0010000
Points: 4
Interpolation: Enabled
Interval: 0.000100
Averaging: Disabled

No.	P/V	波长(nm)	Abs.	描述
1		198. 40	0. 554	
2		194. 80	0. 734	
3		191. 80	5. 000	

页 1 / 1

Figure S11. UV spectrum of **11**.

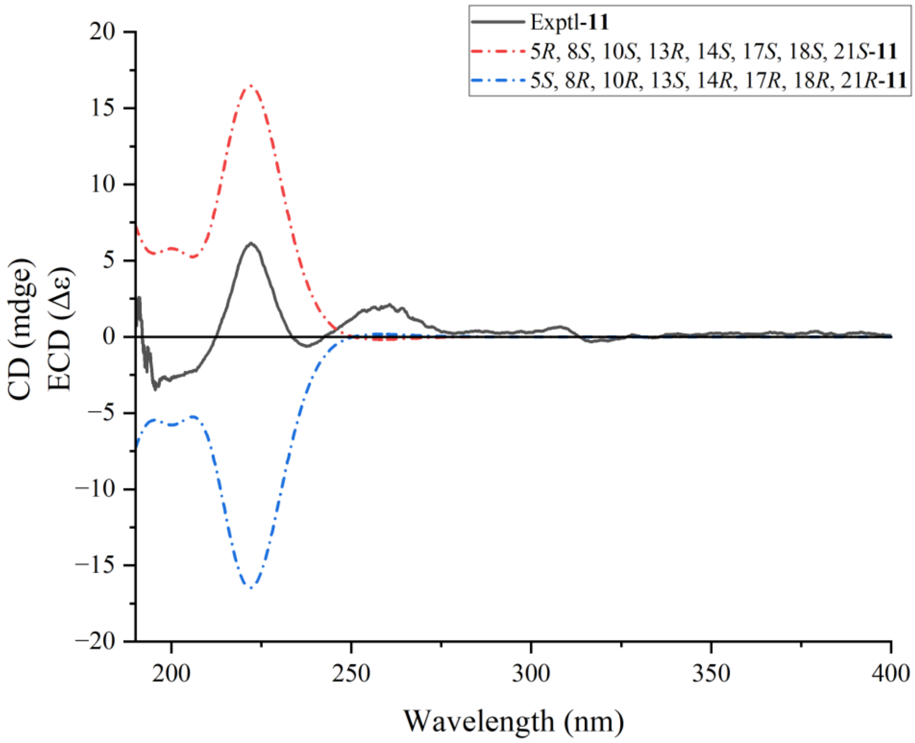

Figure S12. Calculated and experimental ECD spectra for **11**.

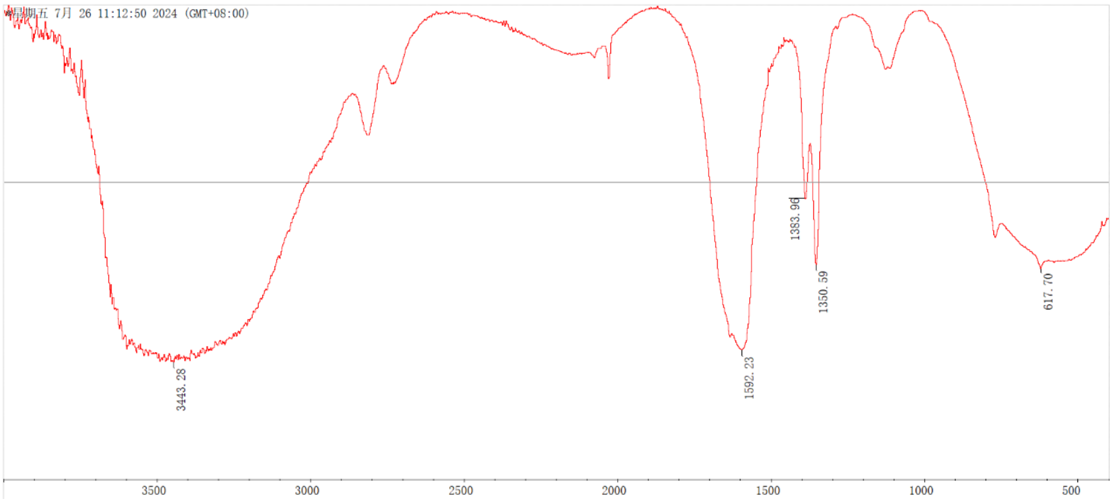

Figure S13. IR spectrum of **11**.

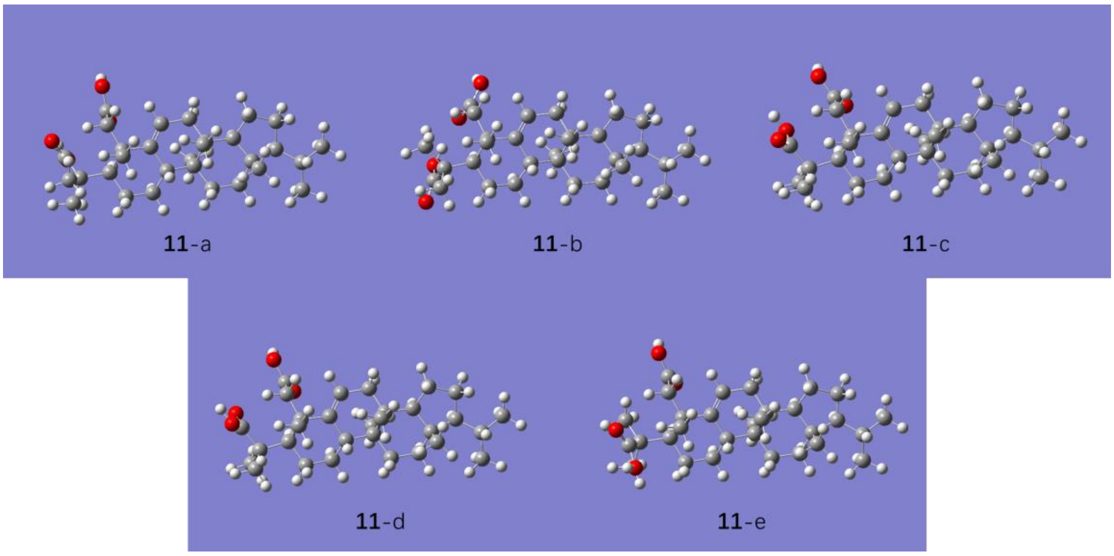

Figure S14. Conformers of isomer **11**.

Table S1. Important thermodynamic parameters and conformational analysis of **11** at the B3LYP/6-311++G(2d,2p) level with CPCM solvent model in methanol.

Conformations	Energy (a.u)	Δ G (kcal/mol)	Boltzmann distributions (%)
11-a	-1473.47268031	1.661075446	3.20
11-b	-1473.47532740	0	52.95
11-c	-1473.47475376	0.359964836	28.82
11-d	-1473.47393520	0.873619422	12.10
11-e	-1473.47259694	1.713390955	2.93

Table S2. Cartesian coordinates for the reoptimized conformers of **11** at the B3LYP/6-311++G(2d,2p) level with CPCM solvent model in methanol.

11-a			Coordinates (Ångstroms)		
Center Number	Atomic Number	Atomic Type	X	Y	Z
1	6	0	-4.889309	-1.255814	0.077603
2	6	0	-3.319659	-0.960225	-0.035807
3	6	0	-2.668297	0.209159	0.825937
4	6	0	-3.441317	1.548221	0.713798

5	6	0	-2.475905	-2.240051	0.137917
6	6	0	-1.04608	-2.021518	-0.360491
7	6	0	-0.30953	-0.879324	0.365475
8	6	0	-1.175513	0.385929	0.42236
9	6	0	1.1196	-0.59921	-0.216314
10	6	0	1.724968	0.650466	0.546249
11	6	0	0.865291	1.871353	0.150547
12	6	0	-0.613031	1.600607	0.296287
13	6	0	2.059111	-1.818357	-0.017608
14	6	0	3.517086	-1.577124	-0.475447
15	6	0	4.171503	-0.366955	0.227409
16	6	0	3.191603	0.844074	0.082169
17	6	0	5.426572	0.261084	-0.481816
18	6	0	5.444434	1.731864	0.054108
19	6	0	4.010601	2.053752	0.561693
20	6	0	-2.635023	-0.097932	2.35629
21	6	0	1.617356	0.510507	2.088354
22	6	0	4.54407	-0.745483	1.679047
23	6	0	6.810571	-0.421409	-0.367623
24	6	0	6.831044	-1.871492	-0.878196
25	6	0	7.876564	0.392599	-1.126303
26	6	0	0.985196	-0.335629	-1.743792
27	6	0	-5.452083	-1.352711	1.50817
28	6	0	-5.238971	-2.594781	-0.634911
29	1	0	3.12568	0.988545	-1.004544
30	1	0	-3.182412	-0.651193	-1.077666
31	1	0	-0.171173	-1.224951	1.401772
32	1	0	1.122786	2.730738	0.786121
33	1	0	1.094625	2.193285	-0.876516

34	1	0	-1.099899	-1.811901	-1.435201
35	1	0	-0.47979	-2.953328	-0.250151
36	1	0	7.103498	-0.4307	0.692482
37	1	0	5.185259	0.295116	-1.556306
38	1	0	3.535101	-1.417962	-1.562067
39	1	0	4.087497	-2.495181	-0.293222
40	6	0	-3.484067	2.243833	-0.630223
41	8	0	-3.362471	1.742407	-1.730489
42	8	0	-3.724324	3.568876	-0.473999
43	6	0	-5.676834	-0.177861	-0.68126
44	8	0	-6.377704	0.684598	-0.18112
45	8	0	-5.551229	-0.290232	-2.020238
46	1	0	-4.484948	1.397119	1.006169
47	1	0	-3.045541	2.263366	1.440716
48	1	0	-2.89913	-3.056472	-0.449749
49	1	0	-2.476865	-2.582667	1.180149
50	1	0	-1.222488	2.498167	0.35604
51	1	0	1.667313	-2.677331	-0.575196
52	1	0	2.053246	-2.123015	1.036843
53	1	0	6.176022	1.830523	0.865484
54	1	0	5.756911	2.429052	-0.729942
55	1	0	3.99773	2.153809	1.652816
56	1	0	3.629662	2.996376	0.154199
57	1	0	-2.175518	-1.062603	2.581269
58	1	0	-2.041976	0.672139	2.860535
59	1	0	-3.630076	-0.090708	2.803259
60	1	0	0.606269	0.743838	2.432232
61	1	0	1.861469	-0.488189	2.45442
62	1	0	2.294895	1.214456	2.582189

63	1	0	4.877672	0.115945	2.26709
64	1	0	3.716396	-1.20886	2.218112
65	1	0	5.362909	-1.473116	1.674595
66	1	0	7.85324	-2.269251	-0.860242
67	1	0	6.209737	-2.537034	-0.272563
68	1	0	6.472008	-1.929071	-1.914076
69	1	0	8.859804	-0.08583	-1.042046
70	1	0	7.969912	1.413508	-0.742612
71	1	0	7.630168	0.457541	-2.19477
72	1	0	0.835607	-1.279466	-2.27812
73	1	0	0.130421	0.30801	-1.969844
74	1	0	1.872188	0.129036	-2.178731
75	1	0	-5.492466	-0.387809	2.014709
76	1	0	-4.852981	-2.042514	2.109448
77	1	0	-6.476393	-1.740717	1.476375
78	1	0	-4.926436	-3.446934	-0.025912
79	1	0	-6.324144	-2.669849	-0.766946
80	1	0	-4.775776	-2.675579	-1.621319
81	1	0	-3.782882	3.954449	-1.369769
82	1	0	-6.041023	0.456574	-2.414468

11-b			Coordinates (Ångstroms)		
Center Number	Atomic Number	Atomic Type	X	Y	Z
1	6	0	-4.935457	-0.917177	0.408917
2	6	0	-3.394863	-0.580462	0.115808
3	6	0	-2.658737	0.61289	0.840079
4	6	0	-3.344631	1.986562	0.542492
5	6	0	-2.57919	-1.88577	0.222696

6	6	0	-1.192272	-1.729953	-0.38551
7	6	0	-0.37361	-0.65845	0.358176
8	6	0	-1.15409	0.663256	0.413111
9	6	0	1.075738	-0.486816	-0.215289
10	6	0	1.760101	0.734686	0.521579
11	6	0	0.984247	1.996642	0.089065
12	6	0	-0.509181	1.831502	0.237007
13	6	0	1.928432	-1.762313	0.020983
14	6	0	3.40225	-1.632497	-0.432399
15	6	0	4.133888	-0.452168	0.244589
16	6	0	3.238664	0.818285	0.0633
17	6	0	5.431413	0.072647	-0.472115
18	6	0	5.545726	1.551724	0.028186
19	6	0	4.134604	1.981459	0.519195
20	6	0	-2.684631	0.45962	2.384432
21	6	0	1.6349	0.638116	2.065692
22	6	0	4.47384	-0.819161	1.707296
23	6	0	6.76601	-0.697651	-0.333124
24	6	0	6.692514	-2.157025	-0.811642
25	6	0	7.888198	0.026018	-1.102308
26	6	0	0.970257	-0.254295	-1.749935
27	6	0	-5.920267	0.251773	0.700448
28	6	0	-5.12634	-1.931184	1.557248
29	1	0	3.188166	0.939961	-1.026955
30	1	0	-3.340635	-0.309156	-0.94079
31	1	0	-0.270972	-1.026542	1.391524
32	1	0	1.292381	2.855339	0.702761
33	1	0	1.236247	2.276633	-0.944693
34	1	0	-1.300253	-1.46474	-1.443845

35	1	0	-0.667866	-2.691311	-0.350653
36	1	0	7.051857	-0.702572	0.728941
37	1	0	5.197728	0.096393	-1.548553
38	1	0	3.437403	-1.503355	-1.522493
39	1	0	3.907358	-2.582305	-0.222475
40	6	0	-3.534833	2.36916	-0.908272
41	8	0	-4.151934	1.731014	-1.754245
42	8	0	-2.99447	3.55965	-1.207383
43	6	0	-5.463523	-1.554662	-0.897793
44	8	0	-5.897179	-2.686369	-0.987075
45	8	0	-5.462901	-0.765698	-1.994504
46	1	0	-4.338512	1.990988	0.987519
47	1	0	-2.798761	2.784641	1.045409
48	1	0	-3.113935	-2.685852	-0.303148
49	1	0	-2.488532	-2.210203	1.265938
50	1	0	-1.049947	2.770858	0.253692
51	1	0	1.482817	-2.607528	-0.516786
52	1	0	1.896496	-2.037733	1.082812
53	1	0	6.278763	1.620989	0.841242
54	1	0	5.907583	2.206856	-0.770719
55	1	0	4.123477	2.108397	1.607518
56	1	0	3.81989	2.93744	0.087017
57	1	0	-3.708151	0.463617	2.772487
58	1	0	-2.208189	-0.469855	2.708073

11-c			Coordinates (Ångstroms)		
Center Number	Atomic Number	Atomic Type	X	Y	Z
1	6	0	-4.837301	-1.370171	-0.020813

2	6	0	-3.323269	-0.901349	-0.252152
3	6	0	-2.677048	0.247389	0.63845
4	6	0	-3.426175	1.594211	0.472176
5	6	0	-2.416269	-2.148164	-0.252397
6	6	0	-1.026969	-1.832733	-0.791824
7	6	0	-0.298022	-0.830645	0.119961
8	6	0	-1.155134	0.427097	0.317003
9	6	0	1.158874	-0.505222	-0.357539
10	6	0	1.748075	0.620766	0.585667
11	6	0	0.920383	1.895256	0.317622
12	6	0	-0.566148	1.635527	0.359881
13	6	0	2.071478	-1.758447	-0.279205
14	6	0	3.551277	-1.488832	-0.642214
15	6	0	4.191971	-0.391241	0.236305
16	6	0	3.235032	0.846193	0.210148
17	6	0	5.483658	0.299784	-0.335019
18	6	0	5.49839	1.688338	0.387985
19	6	0	4.048726	1.968542	0.874939
20	6	0	-2.763987	-0.07674	2.157117
21	6	0	1.571842	0.282047	2.090293
22	6	0	4.498811	-0.960532	1.640357
23	6	0	6.852625	-0.416939	-0.255228
24	6	0	6.876096	-1.788181	-0.950281
25	6	0	7.960561	0.469172	-0.856743
26	6	0	1.093558	-0.047655	-1.842779
27	6	0	-5.0313	-2.344608	1.161265
28	6	0	-5.320806	-2.096411	-1.311037
29	1	0	3.216041	1.130796	-0.850456
30	1	0	-3.307577	-0.502575	-1.271664

31	1	0	-0.212617	-1.331358	1.098014
32	1	0	1.156414	2.66033	1.071407
33	1	0	1.199513	2.345985	-0.646906
34	1	0	-1.123456	-1.433006	-1.808172
35	1	0	-0.446936	-2.759293	-0.867204
36	1	0	7.09989	-0.56979	0.805527
37	1	0	5.288118	0.476977	-1.404706
38	1	0	3.61848	-1.192887	-1.697772
39	1	0	4.100313	-2.433595	-0.555919
40	6	0	-3.539953	2.187541	-0.916434
41	8	0	-2.999945	1.813754	-1.937968
42	8	0	-4.353658	3.272729	-0.89551
43	6	0	-5.800622	-0.201224	0.181976
44	8	0	-6.385472	0.070345	1.214628
45	8	0	-5.983886	0.53015	-0.943762
46	1	0	-4.432439	1.527076	0.886657
47	1	0	-2.954694	2.357117	1.101309
48	1	0	-2.863174	-2.935698	-0.866221
49	1	0	-2.325144	-2.562589	0.759051
50	1	0	-1.159532	2.536217	0.48209
51	1	0	1.693169	-2.532049	-0.957885
52	1	0	2.016378	-2.194722	0.726322
53	1	0	6.196234	1.667357	1.234046
54	1	0	5.85305	2.475154	-0.285516
55	1	0	3.990867	1.927186	1.96841
56	1	0	3.697748	2.962744	0.578106
57	1	0	-3.802877	-0.091352	2.500023
58	1	0	-2.235404	0.692905	2.729223
59	1	0	-2.310419	-1.041295	2.401271

60	1	0	0.548154	0.48147	2.417891
61	1	0	1.792381	-0.758868	2.332498
62	1	0	2.232249	0.906858	2.700266
63	1	0	4.818876	-0.188574	2.348022
64	1	0	3.643384	-1.474261	2.081535
65	1	0	5.307507	-1.696482	1.574078
66	1	0	7.89102	-2.204451	-0.941583
67	1	0	6.219489	-2.514934	-0.463995
68	1	0	6.562602	-1.703136	-1.998987
69	1	0	8.932944	-0.034386	-0.796196
70	1	0	8.050895	1.429322	-0.33892
71	1	0	7.760809	0.677637	-1.916542
72	1	0	0.932729	-0.912792	-2.494414
73	1	0	0.269753	0.64993	-2.015996
74	1	0	2.01191	0.433059	-2.185623
75	1	0	-4.450938	-3.256644	1.003251
76	1	0	-6.085352	-2.631501	1.233184
77	1	0	-4.742119	-1.915299	2.120633
78	1	0	-4.799014	-3.0476	-1.440906
79	1	0	-6.39113	-2.32124	-1.241557
80	1	0	-5.160628	-1.485959	-2.202998
81	1	0	-4.381353	3.623326	-1.806664
82	1	0	-6.602532	1.252454	-0.719994

11-d			Coordinates (Ångstroms)		
Center Number	Atomic Number	Atomic Type	X	Y	Z
1	6	0	-4.847352	-1.356115	-0.074304
2	6	0	-3.32174	-0.90485	-0.266546

3	6	0	-2.684553	0.250108	0.621005
4	6	0	-3.420841	1.601203	0.440211
5	6	0	-2.424877	-2.158292	-0.221346
6	6	0	-1.026005	-1.865009	-0.748869
7	6	0	-0.302473	-0.837935	0.139569
8	6	0	-1.161173	0.423461	0.304698
9	6	0	1.154466	-0.521402	-0.343927
10	6	0	1.741489	0.626333	0.574191
11	6	0	0.91305	1.893666	0.275612
12	6	0	-0.573429	1.633485	0.320983
13	6	0	2.068325	-1.771505	-0.2372
14	6	0	3.547897	-1.508217	-0.60511
15	6	0	4.186731	-0.391186	0.249895
16	6	0	3.228822	0.84457	0.195751
17	6	0	5.478422	0.288263	-0.3351
18	6	0	5.491517	1.691967	0.357893
19	6	0	4.040966	1.981929	0.83664
20	6	0	-2.781675	-0.063689	2.141403
21	6	0	1.563292	0.322176	2.085836
22	6	0	4.49255	-0.929399	1.666373
23	6	0	6.847745	-0.425781	-0.238934
24	6	0	6.872323	-1.811748	-0.904091
25	6	0	7.95557	0.447693	-0.858858
26	6	0	1.089739	-0.096363	-1.839049
27	6	0	-5.072546	-2.341384	1.09485
28	6	0	-5.298192	-2.062489	-1.38486
29	1	0	3.210969	1.10584	-0.870857
30	1	0	-3.277196	-0.519934	-1.289872
31	1	0	-0.217867	-1.313413	1.1301

32	1	0	1.146831	2.675988	1.012169
33	1	0	1.193431	2.322518	-0.69843
34	1	0	-1.107187	-1.49741	-1.778499
35	1	0	-0.449082	-2.795778	-0.787388
36	1	0	7.094453	-0.555472	0.825035
37	1	0	5.283545	0.442238	-1.408494
38	1	0	3.615264	-1.234964	-1.66677
39	1	0	4.097985	-2.450246	-0.498192
40	6	0	-3.491503	2.202413	-0.948433
41	8	0	-3.067358	1.73974	-1.988599
42	8	0	-4.115103	3.404571	-0.905029
43	6	0	-5.796481	-0.160188	0.036595
44	8	0	-6.112379	0.565235	-0.888659
45	8	0	-6.302795	0.028973	1.278964
46	1	0	-4.442372	1.538527	0.816134
47	1	0	-2.968135	2.35584	1.091417
48	1	0	-2.866151	-2.95868	-0.822299
49	1	0	-2.352676	-2.54909	0.800899
50	1	0	-1.167623	2.536164	0.424024
51	1	0	1.691293	-2.560165	-0.899002
52	1	0	2.012861	-2.185619	0.777653
53	1	0	6.188087	1.689337	1.205271
54	1	0	5.846974	2.464343	-0.331699
55	1	0	3.981747	1.964837	1.930676
56	1	0	3.689689	2.969052	0.517357
57	1	0	-2.323773	-1.023201	2.3964
58	1	0	-2.261981	0.71293	2.712417
59	1	0	-3.822796	-0.082879	2.477227
60	1	0	0.538464	0.526376	2.406944

61	1	0	1.786251	-0.712217	2.352616
62	1	0	2.220868	0.962893	2.682221
63	1	0	4.810965	-0.141834	2.357397
64	1	0	3.637235	-1.434448	2.117743
65	1	0	5.302128	-1.665738	1.617105
66	1	0	7.887501	-2.2271	-0.886026
67	1	0	6.216004	-2.528205	-0.402402
68	1	0	6.559147	-1.749635	-1.954498
69	1	0	8.928104	-0.054111	-0.787157
70	1	0	8.045394	1.418752	-0.361685
71	1	0	7.756185	0.63333	-1.922962
72	1	0	0.937523	-0.976189	-2.472794
73	1	0	0.260911	0.591041	-2.029127
74	1	0	2.00517	0.384306	-2.189643
75	1	0	-4.765403	-1.939942	2.061229
76	1	0	-4.518767	-3.2671	0.919304
77	1	0	-6.133264	-2.602919	1.165296
78	1	0	-4.720528	-2.972214	-1.562917
79	1	0	-6.352595	-2.35279	-1.314808
80	1	0	-5.187095	-1.399039	-2.245677
81	1	0	-4.141361	3.7433	-1.82066
82	1	0	-6.908444	0.794285	1.222533

11-e			Coordinates (Ångstroms)		
Center Number	Atomic Number	Atomic Type	X	Y	Z
1	6	0	-4.936851	-0.934209	-0.528825
2	6	0	-3.348367	-0.697261	-0.524525
3	6	0	-2.703947	0.334645	0.503304

4	6	0	-3.423733	1.703333	0.508362
5	6	0	-2.540228	-2.010852	-0.493435
6	6	0	-1.082908	-1.771314	-0.887632
7	6	0	-0.356186	-0.779779	0.039707
8	6	0	-1.182367	0.500458	0.213586
9	6	0	1.118441	-0.486825	-0.40619
10	6	0	1.710765	0.628233	0.549858
11	6	0	0.919754	1.923292	0.262521
12	6	0	-0.573045	1.697204	0.276105
13	6	0	2.001342	-1.759444	-0.308013
14	6	0	3.494362	-1.522478	-0.63742
15	6	0	4.138893	-0.439737	0.256657
16	6	0	3.211055	0.819181	0.208913
17	6	0	5.458867	0.221671	-0.284222
18	6	0	5.488272	1.609884	0.438864
19	6	0	4.034567	1.922997	0.892515
20	6	0	-2.786136	-0.154703	1.982946
21	6	0	1.493879	0.295686	2.050338
22	6	0	4.39985	-1.015856	1.667248
23	6	0	6.809061	-0.525596	-0.172404
24	6	0	6.817828	-1.897946	-0.865687
25	6	0	7.950322	0.334497	-0.748885
26	6	0	1.098897	-0.024663	-1.891702
27	6	0	-5.294188	-2.212241	-1.339447
28	6	0	-5.699342	0.214293	-1.232078
29	1	0	3.223803	1.103839	-0.851674
30	1	0	-3.17068	-0.256715	-1.512251
31	1	0	-0.303104	-1.27284	1.023082
32	1	0	1.159581	2.683264	1.020093

33	1	0	1.229879	2.364726	-0.696711
34	1	0	-1.073162	-1.39935	-1.918939
35	1	0	-0.55037	-2.729258	-0.894285
36	1	0	7.028317	-0.682622	0.893883
37	1	0	5.292429	0.402601	-1.358194
38	1	0	3.591533	-1.227151	-1.690848
39	1	0	4.020643	-2.479061	-0.540093
40	6	0	-3.442357	2.509362	-0.774287
41	8	0	-3.084028	2.161681	-1.881885
42	8	0	-3.953807	3.743454	-0.544623
43	6	0	-5.514214	-1.106275	0.874574
44	8	0	-6.206153	-0.293054	1.461527
45	8	0	-5.190366	-2.291915	1.439179
46	1	0	-4.461512	1.567328	0.826713
47	1	0	-2.991112	2.345472	1.283034
48	1	0	-2.952809	-2.721588	-1.211483
49	1	0	-2.605015	-2.495134	0.48791
50	1	0	-1.15424	2.606133	0.402858
51	1	0	1.620329	-2.523935	-0.995553
52	1	0	1.913774	-2.194645	0.695733
53	1	0	6.165859	1.573516	1.300722
54	1	0	5.876026	2.38814	-0.226276
55	1	0	3.95088	1.883386	1.984361
56	1	0	3.713061	2.924775	0.587879
57	1	0	-3.810724	-0.150882	2.361078
58	1	0	-2.378766	-1.158424	2.117324
59	1	0	-2.206266	0.525216	2.615698
60	1	0	0.468965	0.521106	2.356368
61	1	0	1.682944	-0.750343	2.297337

62	1	0	2.15668	0.90406	2.67416
63	1	0	4.720076	-0.251084	2.382612
64	1	0	3.523163	-1.510669	2.087951
65	1	0	5.193395	-1.769567	1.619857
66	1	0	7.822631	-2.336953	-0.832902
67	1	0	6.133832	-2.609128	-0.39439
68	1	0	6.530962	-1.80723	-1.921516
69	1	0	8.909285	-0.191148	-0.666063
70	1	0	8.050965	1.292812	-0.229545
71	1	0	7.779509	0.546493	-1.813036
72	1	0	0.953819	-0.885368	-2.552618
73	1	0	0.284231	0.678635	-2.085722
74	1	0	2.029109	0.453111	-2.205158
75	1	0	-4.877125	-2.14325	-2.350065
76	1	0	-6.382765	-2.290738	-1.434977
77	1	0	-4.936845	-3.130096	-0.873423
78	1	0	-5.281237	0.377117	-2.229336
79	1	0	-6.753255	-0.05954	-1.347173
80	1	0	-5.680854	1.159108	-0.692597
81	1	0	-3.962987	4.211918	-1.40177
82	1	0	-5.591741	-2.300702	2.329888

The NMR data for known compounds

β-Amyrin ferulate (**1**)

^{1}H NMR (600 MHz, Chloroform-*d*) δ 7.59 (d, *J* = 15.9 Hz, 1H, H-3'), 7.07 (d, *J* = 8.2 Hz, 1H, H-9'), 7.04 (1H, d, J = 1.7Hz, H-5'), 6.91 (d, *J* = 8.2 Hz, 1H, H-8'), 6.29 (d, *J* = 16.0 Hz, 1H, H-2'), 3.93 (s, 3H, 6'-OCH_3), 1.25 (s, 3H, H-27), 1.14 (s, 3H, H-23), 0.99 (s, 3H, H-29), 0.98 (s, 3H, H-30), 0.95 (s, 3H, H-24), 0.92 (s, 3H, H-25), 0.83 (s, 3H, H-26).

^{13}C NMR (151 MHz, Chloroform-*d*) δ 167.3 (C-1'), 147.9 (C-7'), 146.9 (C-6'), 145.4 (C-13), 144.5 (C-3'), 127.3 (C-4'), 123.2 (C-9'), 121.8 (C-12), 116.4 (C-2'), 114.8 (C-8'), 109.4 (C-5'), 80.9 (C-3), 56.1 (-OMe), 55.4 (C-5), 47.7 (C-9), 47.4 (C-18), 46.9 (C-19), 41.9 (C-14), 40.0 (C-8), 38.4 (C-1), 38.1 (C-4), 37.3 (C-22), 37.0 (C-10), 34.9 (C-21), 33.5 (C-29), 32.7 (C-17), 32.6 (C-7), 31.2 (C-20), 28.5 (C-28), 28.2 (C-23), 27.1 (C-16), 26.3 (C-15), 26.1 (C-27), 23.9 (C-30), 23.8 (C-2), 23.7 (C-11), 18.4 (C-6), 17.0 (C-26), 17.0 (C-24), 15.7 (C-25).

Isofouquierol (**2**)

^{1}H NMR (600 MHz, Chloroform-*d*) δ 5.74 (dt, *J* = 15.2, 7.3 Hz, 1H, H-23), 5.61 (d, *J* = 15.9 Hz, 1H, H-24), 3.20 (dd, *J* = 11.5, 4.8 Hz, 1H, H-3), 2.22 (dd, *J* = 13.3, 7.4 Hz, 1H, H-22), 1.12 (s, 3H, H-26), 1.11 (s, 3H, H-27), 0.95 (s, 3H, H-21), 0.94 (s, 3H, H-18), 0.93 (s, 3H, H-19), 0.85 (s, 3H, H-28), 0.83 (s, 3H, H-29), 0.76 (s, 3H, H-30)

^{13}C NMR (151 MHz, Chloroform-*d*) δ137.8 (C-24), 126.9 (C-23), 82.05 (C-3), 79.1 (C-20), 75.5 (C-25), 55.9 (C-5), 50.7 (C-7), 50.4 (C-14), 50.2 (C-17), 43.4 (C-22), 42.6 (C-13), 40.4 (C-8), 39.1 (C-4), 39.0 (C-1), 37.2 (C-10) , 35.3 (C-7), 31.2 (C-15), 28.1 (C-26), 27.6 (C-28), 27.4 (C-27), 25.5 (C-21), 25.1 (C-12), 25.0 (C-16), 24.9 (C-2), 21.6 (C-11), 18.4 (C-6), 16.5 (C-19), 16.3 (C-30), 15.6 (C-29), 15.5 (C-18)

9,19-Cyclolanost-25-ene-3β,24-diol (**3**)

1H NMR (600 MHz, Chloroform-*d*) δ 4.94 – 4.91 (m, 1H, H-26a), 4.83 (dt, *J* = 3.4, 1.7 Hz, 1H, H-26b), 4.02 (t, *J* = 6.5 Hz, 1H, H-24), 3.30 – 3.26 (m, 1H, H-3), 1.72 (s, 3H, H-27), 1.28 (d, *J* = 3.6 Hz, 2H), 1.25 (s, 3H), 0.96 (s, 3H, H-18), 0.88 (d, *J* = 1.7 Hz, 3H, H-21), 0.87 (s, 3H, H-28), 0.80 (s, 3H, H-29), 0.55 (d, *J* = 4.2 Hz, 1H, H-19b), 0.33 (d, *J* = 4.2 Hz, 1H, H-19a).

13C NMR (151 MHz, Chloroform-*d*) δ 147.9 (C-25), 111.1 (C-26), 79.0 (C-3), 76.5 (C-24), 52.3 (C-17), 48.9 (C-14), 48.1 (C-8), 47.2 (C-5), 45.4 (C-13), 40.6 (C-4), 36.0 (C-20), 35.7 (C-12), 33.0 (C-15), 32.1 (C-1), 31.8 (C-22), 30.5 (C-2), 30.0 (C-19), 28.3 (C-23), 28.2 (C-7), 26.6 (C-10), 26.1 (C-11), 25.6 (C-16),21.3 (C-6), 20.1 (C-9), 19.5 (C-21), 18.4 (C-28), 18.2 (C-29), 17.7 (C-18), 17.3 (C-27), 14.1 (C-30).

9,19-Cyclolanost-25-ene-3β,24R-diol (**4**)

^{1}H NMR (600 MHz, Chloroform-*d*) δ 4.94 – 4.91 (m, 1H, H-26a), 4.83 (dt, *J* = 3.4, 1.7 Hz, 1H, H-26b), 4.02 (t, *J* = 6.5 Hz, 1H, H-24), 3.30 – 3.26 (m, 1H, H-3), 1.72 (s, 3H, H-27), 1.28 (d, *J* = 3.6 Hz, 2H), 1.25 (s, 3H), 0.96 (s, 3H, H-18), 0.88 (d, *J* = 1.7 Hz, 3H, H-21), 0.87 (s, 3H, H-28), 0.80 (s, 3H, H-29), 0.55 (d, *J* = 4.2 Hz, 1H, H-19b), 0.33 (d, *J* = 4.2 Hz, 1H, H-19a).

^{13}C NMR (151 MHz, Chloroform-*d*) δ 147.6 (C-25), 111.6(C-26), 79.0 (C-3), 76.5 (C-24), 52.3 (C-17), 48.9 (C-14), 48.1 (C-8), 47.2 (C-5), 45.4 (C-13), 40.6 (C-4), 36.1 (C-20), 35.7

(C-12), 33.0 (C-15), 32.0 (C-1), 31.6 (C-22), 30.5 (C-2), 29.8 (C-19), 28.3 (C-23), 28.2 (C-7), 26.6 (C-10), 26.2 (C-11), 25.6 (C-16),21.3 (C-6), 20.1 (C-9), 19.5 (C-21), 18.5 (C-28), 18.2 (C-29), 17.7 (C-18), 17.3 (C-27), 14.1 (C-30).

Ursolic acid (**5**)

^{1}H NMR (600 MHz, Dimethyl Sulfoxide-*d*$_6$) δ 11.92 (s, 1H, H-COOH), 5.14 (dt, *J* = 17.7, 3.8 Hz, 1H, H-12), 3.00 (dt, *J* = 10.7, 4.3 Hz, 1H, H-3), 2.11 (d, *J* = 11.3 Hz, 1H, H-18), 1.90 (td, *J* = 13.0, 12.5, 3.5 Hz, 2H, H-11), 1.04 (s, 3H, H-27), 0.91 (d, *J* = 6.8Hz 3H, H-30), 0.90 (s, 3H, H-23), 0.87 (s, 3H, H-25), 0.81 (d, *J* = 6.4 Hz, 3H, H-29), 0.72 (s, 3H, H-26), 0.68 (s, 3H, H-24)

^{13}C NMR (151 MHz, Dimethyl Sulfoxide-*d*$_6$) δ 178.3 (C-28), 138.2 (C-13), 124.6 (C-12), 76.8 (C-3), 54.8 (C-5), 52.4 (C-18), 47.0 (C-9), 46.8 (C-16), 41.7 (C-14), 40.1 (C-8), 38.5 (C-20), 38.5 (C-19), 38.4 (C-4), 38.2 (C-1), 36.5 (C-10), 36.3 (C-22), 32.7 (C-15), 30.2 (C-7), 28.3 (C-23), 27.6 (C-21), 27.0 (C-2), 23.8 (C-11), 23.4 (C-27), 23.3 (C-16), 21.1 (C-30), 18.0 (C-6), 17.0 (C-29), 16.9 (C-24), 16.1 (C-25), 15.3 (C-26).

Cycloart-23-ene-3β,25-diol (**6**)

^{1}H NMR (600 MHz, Chloroform-*d*) δ 5.59 (s, 2H, H-23,24), 3.28 (q, *J* = 4.8 Hz, 1H, H-3a), 3.20 (dd, *J* = 11.5, 4.8 Hz, 1H, H-3b), 0.96 (s, 3H, H-18), 0.88 (s, 3H, H-28), 0.80 (s, 3H, H-29), 0.55 (d, *J* = 4.2 Hz, 1H, H-19b), 0.32 (d, *J* = 4.2 Hz, 1H, H-19a)

^{13}C NMR (151 MHz, Chloroform-*d*) δ 139.5 (C-24), 125.8 (C-23), 79.0 (C-3), 70.9 (C-25), 52.2 (C-17), 49.0 (C-14), 48.1 (C-5), 47.2 (C-8), 45.4 (C-13), 40.6 (C-4), 39.2 (C-22), 36.5 (C-20), 35.7 (C-12), 32.9 (C-15), 32.1 (C-15), 30.5 (C-1), 30.3 (C-19), 30.1 (C-26), 30.0 (C-27), 28.2 (C-7), 26.6 (C-16), 26.2 (C-10), 26.1 (C-11), 25.6 (C-29), 21.2 (C-6), 20.1 (C-9), 19.4 (C-28), 18.4 (C-18), 18.2 (C-21), 14.1 (C-30).

11-Oxo-α-amyrin (**7**)

^{1}H NMR (600 MHz, Chloroform-*d*) δ 5.53 (s, 1H, H-12), 3.21 (dd, *J* = 11.4, 5.0 Hz, 1H, H-3), 1.28 (s, 3H, H-27), 1.15 (s, 6H, H-23, 26), 0.99 (s, 3H, H-25), 0.88 (d, *J* = 10.1 Hz, 3H, H-30), 0.78 (s, 3H, H-24).

^{13}C NMR (151 MHz, Chloroform-*d*) δ 200.5 (C-11), 165.1 (C-13), 130.5 (C-12), 78.9 (C-3), 61.6 (C-9), 59.1 (C-18), 55.0 (C-5), 45.3 (C-14), 43.7 (C-8), 41.0 (C-22), 39.4 (C-19), 39.3 (C-20), 39.2 (C-1), 37.2 (C-10), 37.1 (C-4), 34.0 (C-17), 33.0 (C-7), 31.2 (C-21), 28.9 (C-23), 28.2 (C-28), 27.6 (C-2), 27.4 (C-15), 27.3 (C-16), 21.3 (C-30), 20.6 (C-27), 18.8 (C-29), 17.7 (C-26), 17.6 (C-6), 16.6 (C-25), 15.7 (C-24).

11-Oxo-β-amyrin (**8**)

^{1}H NMR (600 MHz, Chloroform-*d*) δ 5.57 (s, 1H, H-12), 3.21 (dd, *J* = 11.4, 5.0 Hz, 1H, H-3), 0.93 (d, *J*=6.8Hz, 3H, H-25), 0.88 (d, *J* = 10.0 Hz, 3H, H-29), 0.80 (s, 3H, H-24), 0.79 (s, 3H, H-26).

^{13}C NMR (151 MHz, Chloroform-*d*) δ 200.0 (C-11), 170.8 (C-13), 128.2 (C-12), 78.9 (C-3), 61.9 (C-9), 55.0 (C-5), 47.7 (C-18), 45.5 (C-19), 45.2 (C-14), 43.5 (C-8), 39.3 (C-1), 39.2 (C-4), 37.1 (C-10), 36.6 (C-22), 34.6 (C-21), 33.2 (C-29), 32.9 (C-7), 32.5 (C-17), 31.0 (C-20),

28.9 (C-28), 28.2 (C-23), 27.4 (C-2), 26.6 (C-15), 26.5 (C-16), 23.6 (C-26), 23.5 (C-26), 18.6 (C-30), 17.6 (C-6), 16.5 (C-24), 15.7 (C-25).

Oleanolic acid (**9**) [33],

^{1}H NMR (600 MHz, Dimethyl Sulfoxide-d_6) δ 11.99 (s, 1H, H-COOH), 5.17 (t, *J* = 3.7 Hz, 1H, H-12), 3.01 (dd, *J* = 10.1, 5.2 Hz, 1H, H-3), 1.10 (s, 3H, H-27), 0.90 (s, 3H, H-26), 0.88 (s, 6H, H-23, 24), 0.86 (s, 3H, H-25), 0.73 (s, 3H, H-29), 0.68 (s, 3H, H-30)

^{13}C NMR (151 MHz, Dimethyl Sulfoxide-d_6) δ 172.0 (C-28), 143.8 (C-13), 121.5 (C-12), 76.8 (C-3), 54.8 (C-5), 47.1 (C-9), 45.7 (C-17), 45.4 (C-19), 41.3 (C-14), 40.8 (C-18), 38.86 (C-8), 38.37 (C-4), 38.04 (C-1), 36.6 (C-10), 32.8 (C-21), 32.40 (C-29), 32.07 (C-22), 31.13 (C-7), 30.4 (C-20), 28.2 (C-23), 26.9 (C-15), 25.6 (C-2), 23.4 (C-27), 22.9 (C-30), 22.6 (C-11), 22.1 (C-16), 18.0 (C-6), 16.8 (C-26), 16.0 (C-24), 15.1 (C-25).

24-methylcycloartane-3β,24,24^{1}-triol (**10**)

^{1}H NMR (600 MHz, Chloroform-*d*) δ 3.52 (dq, *J* = 11.0, 5.7, 4.7 Hz, 2H, H-24^{1}), 3.31 – 3.25 (m, 1H, H-3), 1.00 (s, 3H, H-28), 0.95 (d, *J* = 2.7 Hz, 6H, H-26, 27), 0.88 (s, 3H, H-30), 0.87 (s, 3H, H-29).

^{13}C NMR (151 MHz, Chloroform-*d*) δ 79.0 (C-3), 76.5 (C-24), 71.9 (C-24^{1}), 52.3 (C-17), 48.9 (C-14), 48.1 (C-8), 47.2 (C-5), 45.4 (C-13), 40.6 (C-4), 36.6 (C-20), 35.7 (C-15), 33.0 (C-12), 32.1 (C-25), 32.0 (C-1), 30.5 (C-22), 30.0 (C-2), 29.8 (C-19), 29.5 (C-23), 28.2 (C-16), 26.6 (C-11), 26.2 (C-10), 26.1 (C-7), 25.6 (C-28), 21.2 (C-6), 20.1 (C-9), 19.4 (C-21), 18.5 (C-30), 18.2 (C-18), 17.3 (C-26), 16.6 (C-27), 14.1 (C-29).

β-sitosterol (**12**)

^{1}H NMR (600 MHz, Chloroform-*d*) δ 5.34 – 5.31 (m, 1H), 3.49 (tt, *J* = 11.1, 4.6 Hz, 1H, H-3), 0.99 (s, 3H, H-19), 0.91 (d, *J* = 6.6 Hz, 3H, H-21), 0.83 – 0.81 (m, 3H, H-26), 0.80 (d, *J* = 6.9 Hz, 3H, H-27), 0.66 (s, 3H, H-18).

^{13}C NMR (151 MHz, Chloroform-*d*) δ 37.4 (C-1), 32.0 (C-2), 71.8 (C-3), 42.4 (C-4), 140.9 (C-5), 121.7 (C-5), 32.0 (C-7), 31.7 (C-8), 50.2 (C-9), 36.3 (C-10), 21.2 (C-11), 39.9 (C-12), 42.3 (C-13), 56.8 (C-14), 24.4 (C-15), 28.4 (C-16), 56.1 (C-17), 12.0 (C-18), 19.5 (C-19), 36.6 (C-20), 18.9 (C-21), 34.0 (C-22), 26.1 (C-23), 45.9 (C-24), 29.2 (C-25), 19.9 (C-26), 19.1 (C-27), 23.1 (C-28), 12.1 (C-29).

24-hydroxystigmasta-4,28-dien-3-one (**13**)

^{1}H NMR (600 MHz, Chloroform-*d*) δ 5.84 – 5.76 (m, 1H, H-28), 5.71 (s, 1H, H-4), 5.18 (dd, *J* = 17.2, 4.3 Hz, 1H, H-29a), 5.13 (dd, *J* = 10.8, 3.8 Hz, 1H, H-29b), 1.17 (s, 3H, H-19), 1.02 (s, 1H, H-14), 0.93 (s, 1H, H-9), 0.88 (d, *J* = 2.8 Hz, 3H, H-26), 0.86 (d, *J* = 7.3 Hz, 3H, H-27), 0.69 (s, 3H, H-18)

^{13}C NMR (151 MHz, Chloroform-*d*) δ 199.82 (C-3), 171.83 (C-5), 142.64 (C-28), 123.87 (C-4), 113.01 (C-29), 77.81 (C-24), 56.0 (C-17), 55.9 (C-14), 53.9 (C-9), 42.5 (C-13), 39.7 (C-12), 38.7 (C-10), 36.2 (C-20), 36.0 (C-25), 35.8 (C-1), 35.7 (C-8), 34.9 (C-23), 34.1 (C-2), 33.1 (C-6), 32.1 (C-7), 29.5 (C-22), 28.3 (C-16), 24.3 (C-15), 21.1 (C-11), 18.8 (C-21), 17.7 (C-19), 17.5 (C-26), 16.6 (C-27), 12.1 (C-18).

7β-hydroxysitosterol (**14**)

^{1}H NMR (600 MHz, Chloroform-*d*) δ 5.26 (s, 1H, H-6), 1.02 (s, 3H, H-19), 0.90 (d, *J* = 6.5 Hz, 3H, H-21), 0.86 (d, *J* = 6.7 Hz, 3H, H-29), 0.84 (d, *J* = 6.9 Hz, 6H, H-26,27), 0.67 (s, 3H, H-18).

^{13}C NMR (151 MHz, Chloroform-*d*) δ 143.5 (C-5), 125.5 (C-6), 73.4 (C-7), 71.5 (C-3), 56.0 (C-14), 55.4 (C-17), 48.3 (C-9), 45.9 (C-24), 43.0 (C-12), 41.7 (C-4), 40.8 (C-13), 39.6 (C-8), 37.0 (C-1), 36.5 (C-10), 36.2 (C-20), 34.0 (C-22), 32.0 (C-2), 29.2 (C-23), 28.6 (C-16), 26.4 (C-15), 26.1 (C-25), 23.1 (C-28), 21.2 (C-11), 19.9 (C-27), 19.2 (C-19), 19.1 (C-21), 18.9 (C-26), 12.1 (C-29), 11.9 (C-18).

7α-hydroxysitosterol (**15**)

^{1}H NMR (600 MHz, Chloroform-*d*) δ 5.59 (d, *J* = 5.2 Hz, 1H, H-6), 3.88 – 3.81 (m, 1H), 3.57 (tt, *J* = 10.7, 4.7 Hz, 1H), 0.98 (s, 3H, H-19), 0.92 (d, *J* = 6.5 Hz, 3H, H-21), 0.85 (s, 3H, H-29), 0.83 (d, *J* = 3.2 Hz, 6H, H-26,27), 0.67 (s, 3H, H-18).

^{13}C NMR (151 MHz, Chloroform-*d*) δ 146.4 (C-5), 123.9 (C-6), 71.4 (C-3), 65.5 (C-7), 55.8 (C-17), 49.5 (C-9), 45.9 (C-24), 42.3 (C-12), 42.2 (C-4), 42.1 (C-13), 39.3 (C-8), 37.6 (C-1), 37.5 (C-10), 37.1 (C-8), 36.2 (C-20), 34.0 (C-22), 31.4 (C-2), 29.2 (C-23), 28.4 (C-16), 26.0 (C-15), 24.4 (C-25), 23.2 (C-28), 20.8 (C-11), 19.9 (C-27), 19.1 (C-19), 18.9 (C-21), 18.4 (C-26), 12.1 (C-29), 11.8 (C-18).

Saringosterol (**16**)

^{1}H NMR (600 MHz, Chloroform-*d*) δ 5.80 (dd, *J* = 17.4, 11.0, Hz, 1H, H-28), 5.36 – 5.32 (m, 1H, H-6), 5.18 (d, *J* = 17.3 Hz, 1H, H-29b), 3.52 (qd, *J* = 12.0, 10.7, 6.8 Hz, 1H, H-3), 1.00 (s, 3H, H-19), 0.96 (d, *J* = 2.9 Hz, 3H, H-21), 0.91 (d, *J* = 6.6 Hz, 3H, H-26), 0.87 (d, *J* = 5.8 Hz, 3H, H-27), 0.66 (s, 3H, H-18).

^{13}C NMR (151 MHz, Chloroform-*d*) δ 141.5 (C-28), 139.7 (C-5), 120.7 (C-6), 111.9 (C-29), 77.8 (C-24), 70.8 (C-3), 55.7 (C-14), 55.7 (C-17), 49.1 (C-9), 42.6 (C-13), 41.3 (C-4), 39.5 (C-12), 37.3 (C-1), 36.2 (C-10), 35.5 (C-20), 35.1 (C-25), 31.9 (C-7), 31.4 (C-8), 30.9 (C-23), 30.9 (C-2), 29.3 (C-22), 28.1 (C-16), 24.4 (C-15), 21.7 (C-11), 19.5 (C-19), 19.0 (C-21), 17.8 (C-27), 16.5 (C-26), 10.8 (C-18).

3β-hydroxy-stigmast-5,22-dien-7-one (**17**)

^{1}H NMR (600 MHz, Chloroform-*d*) δ 5.69 (s, 1H, H-6), 5.17 (dd, *J* = 15.2, 8.6 Hz, 1H, H-22), 5.02 (dd, *J* = 15.2, 8.6 Hz, 1H, H-23), 3.67 (tt, *J* = 10.7, 4.6 Hz, 1H, H-3), 1.20 (s, 3H, H-18), 1.02 (d, *J* = 6.6 Hz, 3H, H-21), 0.84 (d, *J* = 6.1 Hz, 3H, H-26), 0.80 (t, *J* = 7.0 Hz, 6H, H-28, 29), 0.69 (s, 3H, H-19).

^{13}C NMR (151 MHz, Chloroform-*d*) δ 202.3 (C-7), 165.1 (C-5), 138.1 (C-22), 129.5 (C-23), 126.1 (C-6), 70.5 (C-3), 54.7 (C-17), 51.2 (C-24), 50.0 (C-14), 49.9 (C-9), 45.4 (C-8), 43.0 (C-13), 41.8 (C-4), 40.3 (C-20), 38.6 (C-12), 38.3 (C-10), 36.4 (C-1), 31.9 (C-25), 31.2 (C-2), 29.1 (C-16), 26.4 (C-15), 25.4 (C-28), 21.4 (C-21), 21.2 (C-11), 21.1 (C-27), 19.0 (C-26), 17.3 (C-19), 12.3 (C-29), 12.2 (C-18).

3β-hydroxystigmast-5-en-7-one (**18**)

^{1}H NMR (600 MHz, Chloroform-*d*) δ 5.65 (d, *J* = 1.8 Hz, 1H, H-6), 3.62 (tt, *J* = 11.2, 4.4 Hz, 1H, H-3), 1.16 (s, 3H, H-19), 0.90 (d, *J* = 6.6 Hz, 3H, H-21), 0.81 (d, *J* = 3.4 Hz, 3H, H-29), 0.80 (d, *J* = 2.7 Hz, 3H, H-27), 0.78 (d, *J* = 6.8 Hz, 3H, H-26), 0.65 (s, 3H, H-18).
^{13}C NMR (151 MHz, Chloroform-*d*) δ 202.7 (C-7), 165.9 (C-5), 126.0 (C-6), 70.4 (C-3), 54.7 (C-17), 50.0 (C-9), 50.0 (C-14), 45.8 (C-24), 45.5 (C-8), 43.2 (C-13), 41.9 (C-4), 38.7 (C-12), 38.4 (C-10), 36.4 (C-1), 36.1 (C-20), 34.0 (C-22), 31.1 (C-2), 29.2 (C-25), 28.6 (C-16), 26.4 (C-15), 26.1 (C-23), 23.1 (C-28), 21.3 (C-11), 19.9 (C-27), 19.1 (C-26), 19.0 (C-21), 17.4 (C-19), 12.0 (C-29), 12.0 (C-18).

Phytyldiol (**19**)
^{1}H NMR (600 MHz, Chloroform-*d*) δ 5.11 (s, 1H, H-17a), 4.94 (s, 1H, H-17b), 4.18 (dd, *J* = 7.6, 3.1 Hz, 2H, H-2), 3.72 – 3.47 (m, 2H, H-1), 1.98 (dddd, *J* = 40.6, 19.6, 10.8, 6.5 Hz, 2H, H-4), 0.86 (s, 3H, H-16), 0.85 (s, 3H, H-20), 0.84 (s, 3H, H-19), 0.83 (s, 3H, H-18)
^{13}C NMR (151 MHz, Chloroform-*d*) δ 148.7 (C-3), 110.4 (C-17), 75.1 (C-2), 65.7 (C-1), 39.4 (C-14), 37.4 (C-8), 37.4 (C-10), 37.3 (C-12), 36.9 (C-6), 33.0 (C-3), 32.8 (C-11), 32.7 (C-7), 28.0 (C-15), 25.5 (C-5), 24.8 (C-13), 24.5 (C-9), 22.7 (C-20), 22.6 (C-16), 19.8 (C-19), 19.7 (C-18).

Caryophyllene oxide (**20**)
^{1}H NMR (600 MHz, Chloroform-*d*) δ 4.97 (s, 1H, H-15a), 4.85 (s, 1H, H-15b), 2.88 (dd, *J* = 10.6, 4.2 Hz, 1H, H-5), 2.61 (q, *J* = 9.4 Hz, 1H, H-9), 1.20 (s, 3H, H-14), 1.00 (s, 3H, H-12), 0.98 (s, 3H, H-13).
^{13}C NMR (151 MHz, Chloroform-*d*) δ 152.0 (C-8), 112.9 (C-15), 63.9 (C-5), 60.0 (C-4), 50.8 (C-1), 48.9 (C-9), 39.9 (C-10), 39.3 (C-3), 34.2 (C-11), 30.3 (C-7), 30.0 (C-6), 29.9 (C-12), 27.3 (C-2), 21.8 (C-13), 17.1 (C-14).
